# Flash Communication: Probing Fe Complex Assembly via Thermogravimetric Analysis

**DOI:** 10.1021/acs.organomet.5c00239

**Published:** 2025-12-12

**Authors:** Jung-Ying Lin, Ernesto R. Lopez, Andrew V. Tran, Raul Bermudes, John Bacsa, Laura K. G. Ackerman-Biegasiewicz

**Affiliations:** Department of Chemistry, 1371 Emory University, Atlanta, Georgia 30322, United States

## Abstract

First-row transition metal catalysis continues to provide innovative and sustainable advances for synthetic chemistry. However, these metals can be challenging to screen efficiently in optimization campaigns due to the limited knowledge of catalyst assembly, stability, and speciation. In this report we demonstrate the use of thermogravimetric analysis (TGA) as a promising tool in evaluating the formation and properties of an Fe precatalyst, *fac*-Fe­(dpa)­Cl_3_. Using TGA it was possible to identify the generation of distinct Fe complexes that could form *in situ* from prestirring the commercial metal salt iron trichloride (FeCl_3_) and di­(2-picolyl)­amine (dpa) in different organic solvents. Upon applying these prestirred mixtures to the reaction between methionine and benzyl acrylate, it was determined that distinct complexes gave distinct TGA profiles. Similar TGA profiles yielded similar reaction yields, while distinct TGA profiles tended to give rise to unique yields. Utilizing this approach, a more informed first-row metal catalyzed reaction strategy can be realized.

## Graphical abstract

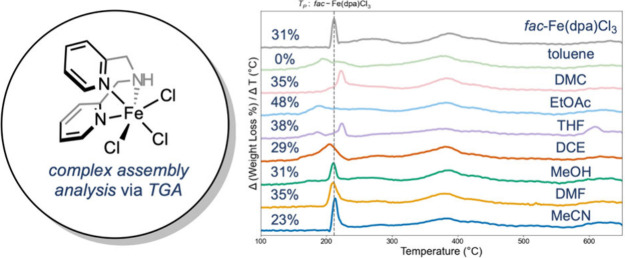

First-row transition metal catalysts have gained prominence as sustainable and complementary alternatives to precious metal catalysts.^1–3^ While they possess unique reactivity, often enabling single electron chemistry, this same advantage can become a limitation in screening and optimization.^4–6^ Unlike well-precedented precious metal catalyst reactions, the selection of a first-row metal catalyst for reactions can be challenging due to the limited number of precedented ligand platforms and the propensity of these catalysts to generate various off-cycle species.^7–10^ Traditional approaches to developing first-row metal catalyzed reactions involve extensive mechanistic exploration of catalytic species, requiring access to well-defined complexes (Figure 1a).^11–13^ However, the synthesis and characterization of first-row transition metal complexes can be difficult and time-consuming, yielding varied success in reactions.^14,15^ An alternative strategy places an emphasis on the efficiency of screening catalysts. It subjects commercially available metal precursors with organic ligands under diverse conditions and then introduces these solutions to reactions to quickly evaluate a breadth of reactivity.^16^ A drawback of this approach is that when reactions fail there is little knowledge about the presumed catalyst structure. Even when procedures involve preligation of a ligand to a metal precursor prior to introduction of reaction components, successful ligation is often assumed rather than verified.^17,18^ As a result, effective methods to probe precatalyst assembly prior to screening are needed.

**Figure 1:**
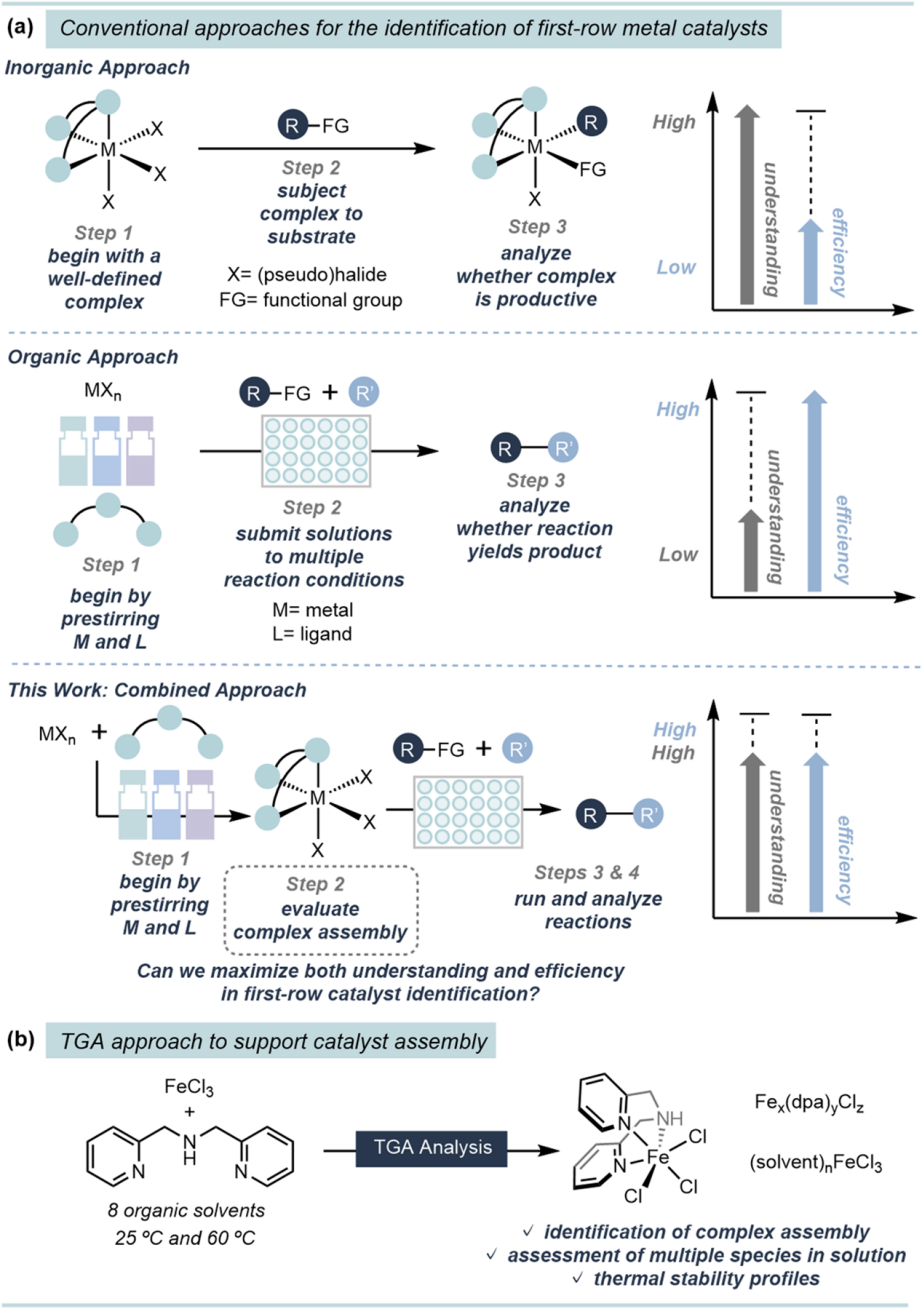
Catalyst assembly in reaction development. (a) Employing stoichiometric vs catalytic amounts of Fe for reaction discovery. (b) TGA analysis for validation of complexation.

While numerous methods are available to study ligation, there are few methods that are competent at assessing catalyst stability and speciation.^19,20^ Additionally, analyzing a large number of catalyst solutions requires a technique that is nonspecialized, efficient and compatible with a wide range of conditions. To meet this need our lab has investigated the use of thermogravimetric analysis (TGA) for catalyst screening as a tool to gain information about precatalyst formation. While seldom used for catalyst optimization in organic chemistry, TGA is a technique which has been widely applied in material chemistry to assess the composition and degradation of solid materials such as polymers, metal organic frameworks, and nanoparticles.^21–23^ It has also been used as a standard characterization technique for isolated, well-defined inorganic complexes and in the determination of thermal stabilities.^24^ With this established precedent, we envisioned repurposing solid sample TGA analysis to evaluate solutions of metal complexes that had been formed *in situ* (Figure 1b). We imagined this strategy would offer a straightforward procedure to validate precatalyst formation which would in turn give insight into reaction design.

Given the persistent interest in the development of Fe catalysis as a sustainable approach to organic chemistry,^25–35^ this study focused on the evaluation of a precedented Fe precatalyst for visible light-induced homolysis (VLIH), *fac*-Fe­(dpa)­Cl_3_.^36–38^ In a typical reaction screen FeCl_3_ and di­(2-picolyl)­amine (dpa) would be prestirred together in an organic solvent, with the formation of Fe­(dpa)­Cl_3_ unverified. Therefore, we began our analysis by investigating whether Fe­(dpa)­Cl_3_ could be observed by TGA upon prestirring FeCl_3_ and dpa in 8 commonly used organic solvents and at two commonly used prestirring temperatures, 25 and 60 °C.

To provide a standard TGA profile of *fac*-Fe­(dpa)­Cl_3_ for comparison with the crude solution, the presynthesized complex was subjected to TGA with a temperature ramp rate of 10 °C/min from 25 to 650 °C. It was observed that *fac*-Fe­(dpa)­Cl_3_ as a solid was thermally stable with nearly 100% weight retained up to 200 °C (Figure 2a). When examining the first derivative of the TGA curve of *fac*-Fe­(dpa)­Cl_3_ four unique decomposition peaks were revealed at 211, 270, 391, and 443 °C. In comparison to the degradation patterns of independent samples of FeCl_3_ and dpa, the *fac*-Fe­(dpa)­Cl_3_ thermogravimetric profile showcased a prolonged delay in its peak temperature (*T*_*p*_) at initial degradation (*T*_*p*_ = 211.86 °C). While the weight loss of this degradation did not align with the mass of dpa or chlorine, the evolved substance from this degradation was characterized as 2-methylpyridine by GCMS (Figures S31 and S32).

**Figure 2:**
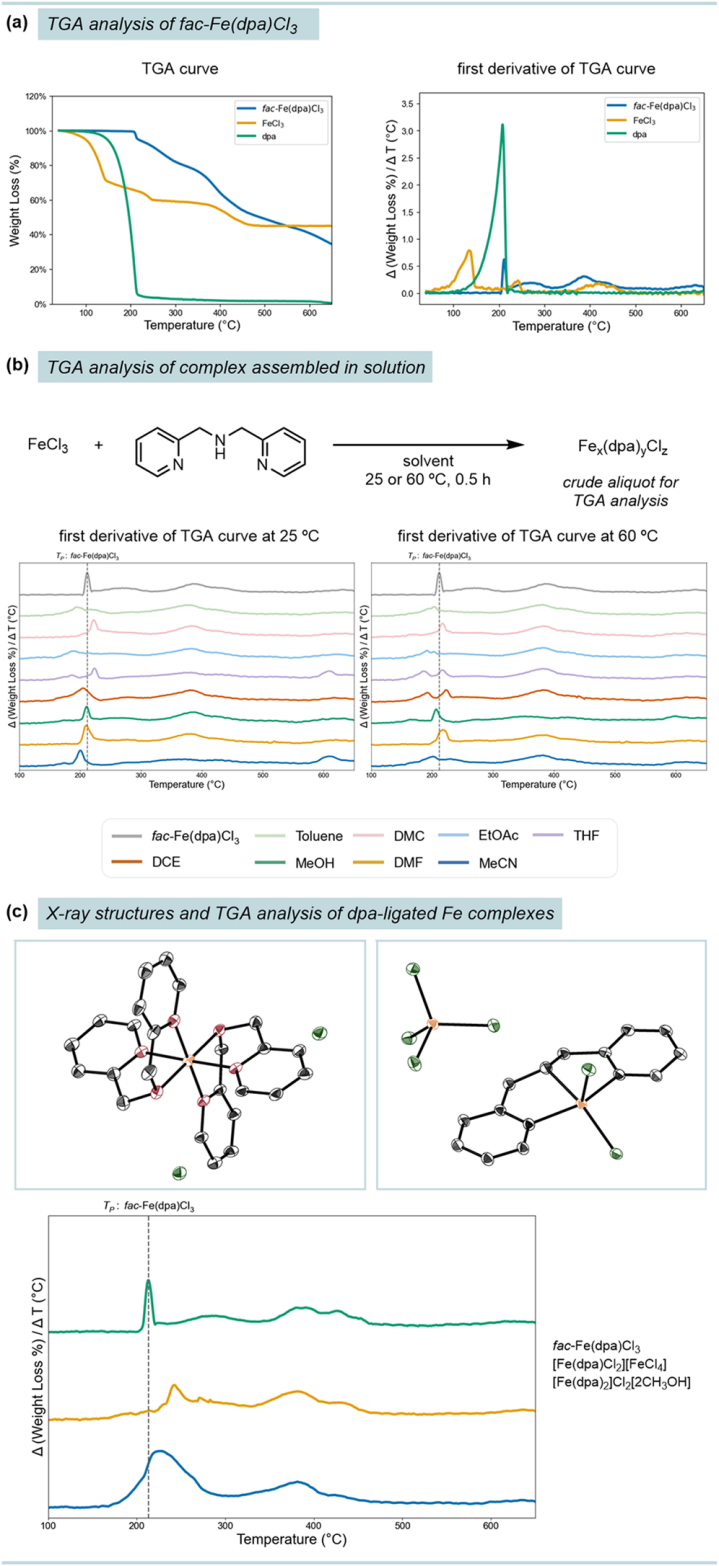
Validation of complexation using thermogravimetric analysis (TGA). (a) Thermal degradation profile of *fac*-Fe­(dpa)­Cl_3_. (b) First derivative of TGA curves depicting solution phase aliquots of FeCl_3_ and dpa stirred in different solvents at the reported reaction temperature. (c) X-ray structures and TGA analysis of [Fe­(dpa)­Cl_2_]­[FeCl_4_] and [Fe­(dpa)_2_]­Cl_2_[2CH_3_OH].

With the well-defined thermal degradation profile of *fac*-Fe­(dpa)­Cl_3_ obtained by TGA, we next evaluated the solutions produced by TGA by stirring FeCl_3_ and dpa in various reaction solvents. We hypothesized that those solvents that yielded *fac*-Fe­(dpa)­Cl_3_ would exhibit similar first derivative TGA profiles as the solid-state sample attained previously. Furthermore, a change in decomposition temperature could indicate a change to the precatalyst or formation of a distinct species. To realize this approach, complex formation was examined in methanol (MeOH), tetrahydrofuran (THF), ethyl acetate (EtOAc), acetonitrile (MeCN), 1,2-dichloroethane (DCE), dimethyl carbonate (DMC), toluene, and *N*,*N*-dimethylformamide (DMF). While stirring FeCl_3_ and dpa, yellow precipitates were clearly observed in all cases which are typically indicative of Fe(dpa)Cl_3_. Aliquots of these stirred mixtures were then subjected to TGA. To minimize solvent-related interference in the TGA measurement while avoiding premature weight loss from low-boiling components, each sample was held at 82 °C for 10 min prior to ramping to 650 °C. For DMF-containing samples, a longer hold was applied to ensure complete solvent removal before the onset of sample degradation (Figure S49).

Upon examination of each of the first derivative TGA plots of the prestirred solutions, a higher *T*_*p*_ at initial degradation than FeCl_3_ and dpa alone was observed (Figures S14–S29). These results suggest that the species resulting from ligation of dpa and FeCl_3_ are thermally more stable than FeCl_3_. Attempts at determining ligation statuses of these FeCl_3_ and dpa mixtures proved challenging with UV–vis spectroscopy (Figure S1–S8). In most of the solvents used in this report, *fac*-Fe­(dpa)­Cl_3_ was insoluble, with the exception of DMF and toluene. As a result, the UV–vis absorbance spectra were not definitive in determining ligation, due to insolubility of the complex or lack of homogeneity of the solution.

Instead, by comparing the *T*_*p*_ at the first degradation of the crude mixture with that of the dry *fac*-Fe­(dpa)­Cl_3_ or *fac*-Fe­(dpa)­Cl_3_ in different solvents, a significant difference of the *T*_*p*_ was observed in most of the solvents, except MeOH and DMF (Figure 2b and Figures S14–S29). We hypothesized these differences could result from solvent interactions with complexes or from speciation differences across solvents. To probe this hypothesis, IR spectroscopy was used to further examine the identity of the resultant yellow precipitates derived from filtration of the crude ligation mixtures. The IR spectra of the solids derived from complexation demonstrated features similar to those of the reported *fac*-Fe­(dpa)­Cl_3_ complex (Figures S33–S35, S38–S40, and S42–S47), which suggests the difference of the observed *T*_*p*_ could result from solvent interactions. In contrast, complexes formed in THF at 25 and 60 °C as well as DCE at 60 °C showcased IR spectra which significantly deviated from *fac*-Fe­(dpa)­Cl_3_, suggesting formation of distinct species from *fac*-Fe­(dpa)­Cl_3_ under these stirring conditions (Figures S36, S37, and S41). To confirm this hypothesis, the filtered yellow precipitate derived from stirring in THF was further submitted to TGA as a solid and possessed an initial *T*_*p*_ of 223.55 °C similar to that of the solution phase sample (*T*_*p*_ = 223.76 °C). This suggested the formation of a unique species which is not solvent dependent (Figure S30). Elemental analysis of this solid indicated an elemental composition similar to that of *fac*-Fe­(dpa)­Cl_3_. Together these data serve as an initial demonstration of the potential for TGA to identify distinct complexes from differences in degradation profiles.

Because the IR spectrum of the mixture formed by stirring FeCl_3_ and dpa in DCE at 60 °C differed from that of *fac*-Fe­(dpa)­Cl_3_, we pursued further characterization of this species. This led to the successful crystallization of [Fe­(dpa)­Cl_2_]­[FeCl_4_] from the filtrate, and was confirmed by X-ray diffraction and elemental analysis (Figure 2c). Additionally, this complex exhibited a unique degradation profile, distinct from those of *fac*-Fe­(dpa)­Cl_3_ with an earlier onset degradation (*T*_*p*_ = 174.88 °C).

To further support the ability of TGA to differentiate mixtures of solids by different degradation profiles, we hypothesized that another potential possible species that could arise from stirring could be a bisligated [Fe­(dpa)_2_]­Cl_2_ complex. We independently synthesized a [Fe­(dpa)_2_]­Cl_2_[2CH_3_OH] complex, whose structure was confirmed by X-ray diffraction and elemental analysis. Upon mixing *fac*-Fe­(dpa)­Cl_3_ with [Fe­(dpa)_2_]­Cl_2_[2CH_3_OH] and analyzing its degradation, a profile distinct from both individual components was observed (Figure S10). [Fe­(dpa)_2_]­Cl_2_[2CH_3_OH] has two degradation events, with a *T*_*p*_ = 214.25 °C and *T*_*p*_ = 271.56 °C respectively. However, a 1:1 mixture of *fac*-Fe­(dpa)­Cl_3_ and [Fe­(dpa)_2_]­Cl_2_[2CH_3_OH] resulted in a distinct TGA profile with *T*_*p*_ = 242.01 °C. These results support the idea that TGA profiles of sample mixtures can yield unique degradation features of distinct from the individual complexes alone.

To evaluate the differences in complex formation suggested by TGA, we benchmarked the catalytic activity of FeCl_3_ and dpa mixtures in a Fe-mediated decarboxylative Giese reaction that proceeded through VLIH (Scheme 1).^26^ Mixtures of FeCl_3_ and dpa stirred in toluene at 25 °C resulted in a 0% yield. However, stirring at 60 °C increased the yield to 16%. This difference was mirrored in the TGA profiles where the mixture stirred at 25 °C displayed an initial degradation *T*_*p*_ at 196 °C with a minor secondary *T*_*p*_ at 217 °C, while the 60 °C mixture exhibited only a single degradation *T*_*p*_ at 202 °C. Similarly, *fac*-Fe­(dpa)­Cl_3_ stirred in various solvents and analyzed by TGA consistently showed a single degradation *T*_*p*_ at 214 °C (Figure S48). Subjecting these complexes to the Giese reaction gave an average yield of 31% with a small standard deviation (±3%) (Table S3), supporting that TGA can distinguish and identify similar species from solution aliquots.

**Scheme 1:**
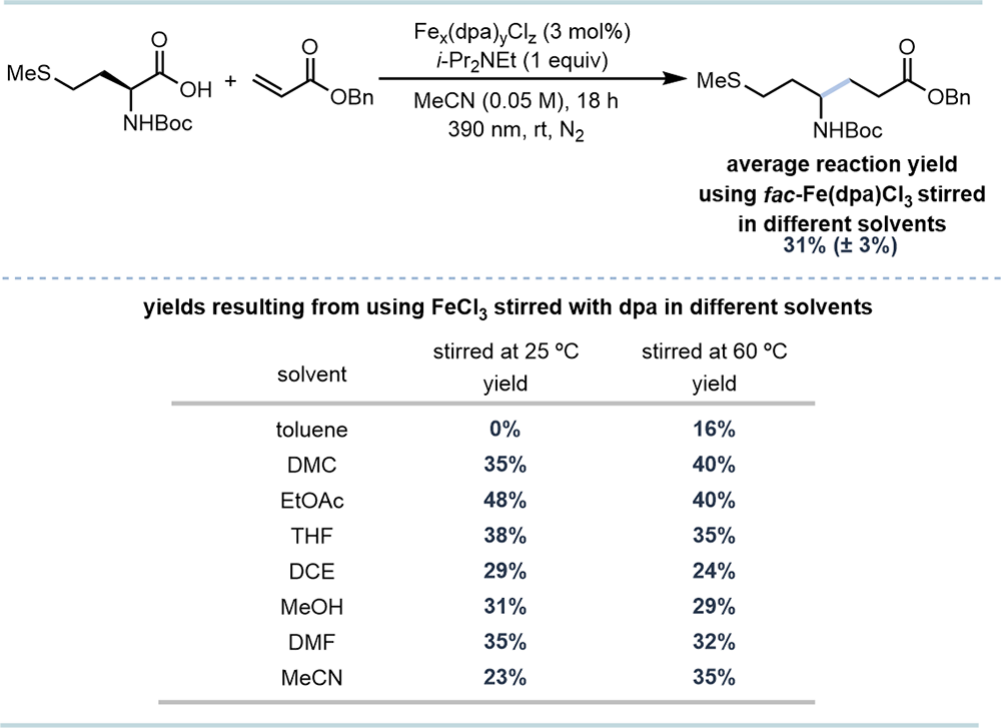
Catalytic Activity of FeCl_3_ and dpa Mixtures in a Fe-Mediated Photodecarboxylative Giese Reaction^a^

a Yields were determined by ^1^H NMR with dibromomethane as an external standard.

In conclusion, we have demonstrated the ability of TGA to analyze Fe complex assembly from prestirred mixtures of metal salts and ligands. While prior methods utilized TGA primarily as a solid-state analytical tool for well-defined inorganic complexes, our workflow demonstrates that TGA can also be utilized to evaluate mixtures of metal complexes taken as aliquots from solutions. Notably, sample aliquots could be assessed without solubility limitations, and thermal degradation profiles provided valuable insight into Fe complex stability and speciation. TGA was able to differentiate between *fac*-Fe­(dpa)­Cl_3_, [Fe­(dpa)_2_]­Cl_2_[2CH_3_OH], and [Fe­(dpa)­Cl_2_]­[FeCl_4_], all of which resulted in giving different yields in an Fe-mediated photodecarboxylative Giese reaction. The results of this report ultimately present a promising analytical workflow to inform reaction development using first-row transition metal catalysts.

## Supplementary Material



## Notes

Experimental procedures, spectroscopy, thermal degradation profiles, and crystallographic details (PDF)
