# Supplementary material for: Flash Communication: Probing Fe Complex Assembly via Thermogravimetric Analysis
Source: Organometallics. 2025 Dec 12;45(2):111–6. doi: 10.1021/acs.organomet.5c00239 (PMC12848960; doi:10.1021/acs.organomet.5c00239)
Supplement: Supplementary file 1 [file om5c00239_si_001.pdf]

Supporting Information for Flash Communication: Probing Fe Complex Assembly via Thermogravimetric Analysis

Jung-Ying Lin, Ernesto R. Lopez, Andrew V. Tran, Raul Bermudes Jr., John Bacsa, Laura K. G. Ackerman-Biegasiewicz*

Department of Chemistry, Emory University, Atlanta, GA 30322, United States

laura.ackerman@emory.edu

Table of Contents

I. General Information. 3

II. Experimental Procedures..5

1. Synthesis of *fac*-Fe(dpa)Cl_3 complex. 5

2. Synthesis of [Fe(dpa)$_2$]Cl_2[2CH_3OH] complex. 5

3. Procedure for the mixture of $FeCl_3$ and dpa in different solvents at room temperature. 6

4. Procedure for the mixture of $FeCl_3$ and dpa in different solvents at 60 °C. 6

5. Procedure for *fac*-Fe(dpa)Cl_3 dissolved in different solvents at different temperatures. 7

6. Isolation of precipitate from the mixture of $FeCl_3$ and dpa. 7

7. Isolation of soluble material from the mixture of $FeCl_3$ and dpa in DCE. 7

8. General procedure for Fe mediated photodecarboxylative Giese reaction. 8

III. UV-Vis spectroscopy. 9

1. UV-Vis sample preparation. 9

2. UV-Vis spectra 9

IV. TGA analysis. 12

1. TGA procedure..12

2. Reproducibility studies of TGA for *fac*-Fe(dpa)Cl_3 samples...14

3. TGA Analysis of mixtures of *fac*-Fe(dpa)Cl_3 and [Fe(dpa)$_2$]Cl_2[2CH_3OH].................................15

4. Overlaid first derivative TGA of Fe(dpa)Cl_3 and mixtures of $FeCl_3$ and dpa in various solvents. 16

5. TGA Profiles of $FeCl_3$ and dpa solutions at 25 °C. 18

6. TGA Profiles of $FeCl_3$ and dpa solutions at 60 °C. 22

V. Characterization of *fac*-Fe(dpa)Cl_3 Degradation through Gas Chromatography Mass Spectrometry (GCMS)...27

VI. IR Spectroscopy. 28

VII. Reaction Yields for Fe Mediated Decarboxylative Giese Reaction 34

VIII. Holding Temperature Analysis...35

IX. Percent Mass Loss for TGA Samples..36

X. X-ray data 37

1. X-ray crystal data of [Fe(dpa)$_2$]Cl_2[2CH_3OH]...37

2. X-ray crystal data of $[Fe(dpa)Cl_2]FeCl_4$ 44

XI. References. 50

I. General Information.

Materials. Unless otherwise stated, all reagents are commercial, purchased as reagent grade, and used without further purification. Materials were sourced from Sigma-Aldrich, Oakwood Chemical, and AmBeed. For the synthesis of the *fac*-Fe(dpa)Cl_3 complex, Anhydrous $FeCl_3$ was purchased from Oakwood Chemical, stored in a N_2-filled glovebox, and used as received. dipicolylamine (dpa) was purchased from AmBeed. All purchased anhydrous solvents were used as received.

Methods. Unless otherwise stated, all procedures were conducted on the benchtop. Masses were measured using either an Accuris instruments model W3101A-120 or VWR model VWR-224AC balance.

Instrumentation. Thermogravimetric Analysis (TGA) was performed using Mettler Toledo TGA/DSC 3+ STARe Thermal Analysis System. TGA samples were prepared in 70 μL platinum crucibles (Mettler Toledo, 51119654) and analyzed by TGA under an inert atmosphere (nitrogen). TGA was performed under constant N_2 flow (50 mL/min). After sample insertion, the sample method had a 10-minute hold time at 25 °C to flush the furnace with N_2 and was further heated to 650 °C at a ramp rate of 10 °C/min. To evaporate solvents either a Heidolph Hei–VAP Core or Hei–Vap Ultimate rotary evaporator at 25–35 °C and <50 torr was used. The SP Genevac EZ–2 4.0 from Integrated Scientific Solutions was used to evaporate solvents. GCMS analysis was performed on an Agilent 5977C GC/MSD equipped with an HP-5MS UI column (30 m × 0.25 mm × 0.25 μm) with a quadrupole mass analyzer using helium as the carrier gas. The analysis method used in all cases was 1 μL injection of sample, an injection temp of 250 °C, and a 20:1 split ratio. The initial inlet pressure was 20.792 psi but varied as the column flow was held constant at 1.8 mL/min for the duration of the run. The interface temperature was held at 280 °C, and the ion source (EI+, 70 eV) was held at 280 °C. The initial oven temperature was held at 60 °C for 0.5 min, followed by a temperature ramp to 300 °C at 60 °C/min. The detector was turned on after 1.80 min. The total run time was 6.50 min. Fourier transform infrared (FT-IR) spectra were recorded on a Thermo-Fischer Nicolet iS50 using attenuated total reflectance (ATR) and are reported in terms of frequency of absorption (cm^{-1}). Ultraviolet-Visible (UV-Vis) absorbance spectra were collected in BMG ClarioStar® Plus plate reader. Spectra were recorded from 220 nm to 800 nm with a resolution of 1 nm. Well scanning was performed using a spiral averaging pattern with a 4 mm diameter. The frequency of scans was set at 51 scans per 0.1 second. No shaking of the plate was performed prior to the acquisition. Upon completion of spectral acquisition, the resulting data were accessed using MARS data analysis software. To obtain crystal structures for the Fe complexes a XtaLAB Synergy, Dualflex, and HyPix diffractometer was used. Proton nuclear magnetic spectroscopy (^1H NMR) was recorded on a Bruker spectrometer equipped with a 5 mm iProbe (^1H at 400 MHz). Chemical shifts for protons are reported in parts per million downfield from tetramethylsilane and are referenced to residual protium in the NMR solvent.[1,2] Photocatalytic

reactions were performed using Kessil PR160L lights (purchased from Kessil: https://kessil.com/products/science_main.php) with a maximum absorbance of the specified wavelength at 100% intensity.

II. Experimental Procedures.

1. Synthesis of *fac*-Fe(dpa)Cl_3 complex.

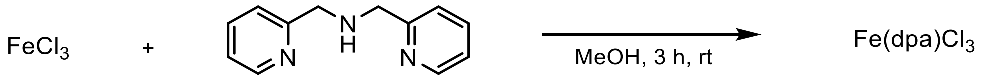

fac-Fe(dpa)Cl_3 was synthesized based on a modified literature procedure.[3] In a 20 mL scintillation vial (Fisherbrand Glass Vial, 03-337-15) equipped with a PTFE-coated stir bar (VWR spinbar micro, 3 × 10 mm, 58948-375), a 0.200 M solution of anhydrous $FeCl_3$ (1.00 mmol, 162.20 mg in 5.00 mL) was prepared in anhydrous methanol. In a separate vial, a 0.200 M solution of dpa (1.00 mmol, 180 μL in 5.00 mL) was prepared. To form the complex, dpa solution was added to the stirring $FeCl_3$ solution and a yellow precipitate was observed, which was left to stir at room temperature for 3 hours. The yellow solid was filtered through a fritted funnel under vacuum and washed with cold anhydrous methanol. The product was dried under vacuum to furnish 303 mg (84% yield) of a yellow solid.

Characterization

FTIR–ATR (cm^{-1}): 3238 (m), 2929 (vw) 1605 (w), 1576 (w), 1488 (w), 1438 (m), 1308 (w), 1278 (w), 1240 (vw), 1161 (vw), 1023 (m), 998 (w), 946 (w), 916 (w), 768 (s), 726 (vw), 643 (s), 474 (vw), 432 (m), 352 (vw), 285 (vs), 235 (vs), 212 (vs), 158 (vs).

Elemental Analysis calculated for [C12H13N3Cl3Fe]: C 39.87%, H 3.63%, N 11.63%, Cl 29.42%; found: C 39.88%, H 3.49%, N 11.49%, Cl 29.50%.

2. Synthesis of [Fe(dpa)$_2$]Cl_2[2CH_3OH] complex.

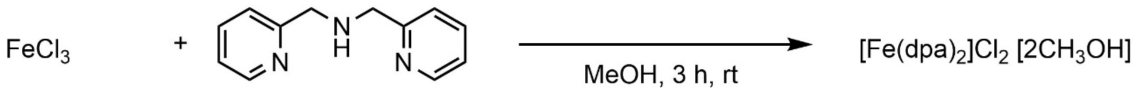

[Fe(dpa)$_2$]Cl_2[2CH_3OH] was synthesized based on the procedure followed for the synthesis of the *fac*-Fe(dpa)Cl_3 with an excess of dpa. In a 20 mL scintillation vial (Fisherbrand Glass Vial, 03-337-15) equipped with a PTFE-coated stir bar (VWR spinbar micro, 3 × 10 mm, 58948-375), a 0.200 M solution of anhydrous $FeCl_3$ (1.00 mmol, 162.20 mg in 5.00 mL) was prepared in anhydrous methanol. In a separate vial, a 0.600 M solution of dpa (3.00 mmol, 520 μL in 5.00 mL) was prepared. To form the complex, dpa solution was added to the stirring $FeCl_3$ solution and a coloration change to dark red and a yellow precipitate of *fac*-Fe(dpa)Cl_3 was

observed. The mixture was stirred at room temperature for 3 hours. The solution was then filtered through PTFE syringe filter (VWR® Syringe Filters, 0.22 µm pore size, 76479-006) into a 100 ml round-bottom flask. To the reaction crude 50 ml of anhydrous diethyl ether was added, and the slurry was stored in the fridge for 20 minutes. The reaction solution was filtered with a fritted funnel under vacuum and the red solid obtained was washed with three portions of 3.00 mL of cold diethyl ether and dried under vacuum to afford $Fe(dpa)_2Cl_2 \cdot 2CH_3OH$ complex $_2$ as a red solid (232.4 mg, 0.40 mmol, 40%). Crystals suitable for X-ray diffraction were obtained from slow evaporation of a concentrated solution of $Fe(dpa)_2Cl_2$ in MeOH.

Characterization

FTIR–ATR (cm^{-1}): 3214 (m), 2925 (vw) 1605 (w), 1566 (w), 1486 (w), 1443 (m), 1311 (w), 1280 (w), 1159 (vw), 1122 (m). 1070 (m), 1021 (m), 968 (w), 909 (w), 819 (w), 768 (s), 726 (vw), 544 (br), 489 (m), 458 (m), 441 (m) 403.5 (m) 358 (s), 312 (s), 292 (sh), 262 (sh).

Elemental Analysis calculated for [C24H32Cl2FeN6O3]: C 52.99%; N 14.26%, H 5.82%; Cl 12.03.52%; found: C 47.60%, N 13.51 %, H 5.54%, Cl 12.14%.

3. Procedure for the mixture of $FeCl_3$ and dpa in different solvents at room temperature.

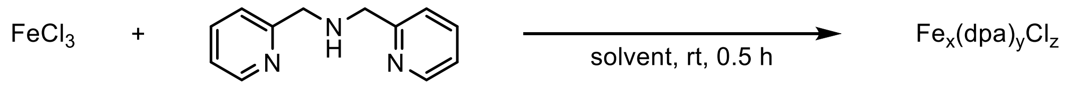

A 1-dram vial (Fisherbrand Class A Glass Vial, 0333925B) equipped with a PTFE-coated stir bar (VWR spinbar micro, 3 × 10 mm, 58948-375) was charged with anhydrous $FeCl_3$ (0.100 mmol, 16.2 mg) and anhydrous solvent (MeOH, MeCN, THF, toluene, DCE, EtOAc, DMC, DMF) (0.500 mL). In a separate vial, a 0.200 M solution of dpa (0.200 mmol, 36.0 µL in 1.00 mL) was prepared. To induce formation of the complex, 0.500 mL dpa solution was added to the stirring $FeCl_3$ solution and was left to react at room temperature for 30 min. An aliquot of the reaction mixture was further analyzed by TGA and UV-Vis spectroscopy.

4. Procedure for the mixture of $FeCl_3$ and dpa in different solvents at 60 °C.

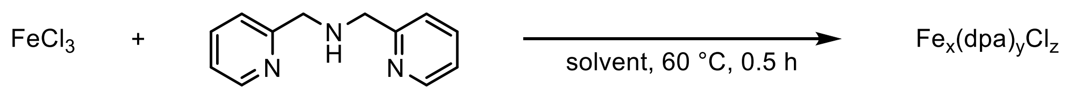

A 1-dram vial (Fisherbrand Class A Glass Vial, 0333925B) equipped with a PTFE-coated stir bar (VWR spinbar micro, 3 × 10 mm, 58948-375) was charged with anhydrous $FeCl_3$ (0.100 mmol, 16.2 mg) and anhydrous solvent (MeOH, MeCN, THF, toluene, DCE, EtOAc, DMC, DMF) (0.500 mL). In a separate vial, a 0.200 M solution of dpa (0.200 mmol, 36.0 μL in 1.00 mL) was prepared. For the formation of the complex, 0.500 mL dpa solution was added to the stirring $FeCl_3$ solution and was left to react at 60 °C for 30 min. An aliquot of the reaction mixture was further analyzed by TGA and UV-Vis spectroscopy.

5. Procedure for *fac*-Fe(dpa)Cl_3 dissolved in different solvents at different temperatures.

A 1-dram vial (Fisherbrand Class A Glass Vial, 0333925B) equipped with a PTFE-coated stir bar (VWR spinbar micro, 3 × 10 mm, 58948-375) was charged with *fac*-Fe(dpa)Cl_3 (0.100 mmol, 36.1 mg) and anhydrous solvent (MeOH, MeCN, THF, toluene, DCE, EtOAc, DMC, DMF) (1.000 mL). The resulting yellow slurry was stirred for 30 minutes at room temperature or at 60 °C. An aliquot of the reaction mixture was further analyzed by thermogravimetric analysis or UV-Vis spectroscopy.

6. Isolation of precipitate from the mixture of $FeCl_3$ and dpa.

A 1-dram vial (Fisherbrand Class A Glass Vial, 0333925B) equipped with a PTFE-coated stir bar (VWR spinbar micro, 3 × 10 mm, 58948-375) was charged with anhydrous $FeCl_3$ (0.100 mmol, 16.2 mg) and anhydrous solvent (0.500 mL). In a separate vial, a 0.200 M solution of dpa (0.200 mmol, 36.0 μL in 1.00 mL) was prepared. For the formation of the precipitate, the dpa solution was added to the stirring $FeCl_3$ solution and was left to react at room temperature or 60 °C for 30 min. The yellow precipitate was filtered off and washed with the corresponding reaction solvent. The resulting product was dried under vacuum and analyzed by TGA and FT-IR spectroscopy.

7. Isolation of soluble material from the mixture of $FeCl_3$ and dpa in DCE.

A 1-dram vial (Fisherbrand Class A Glass Vial, 0333925B) equipped with a PTFE-coated stir bar (VWR spinbar micro, 3 × 10 mm, 58948-375) was charged with anhydrous $FeCl_3$ (0.100 mmol, 16.2 mg) and anhydrous 1,2-dichloroethane (0.500 mL). In a separate vial, a 0.200 M solution of dpa (0.100 mmol, 36.0 μL in 1.00 mL) was prepared. For the formation of the precipitate, the dpa solution was added to the stirring $FeCl_3$ solution and was left to react at room temperature or 60 °C for 2 hours. The solution was then filtered through PTFE syringe filter (VWR® Syringe Filters, 0.22 μm pore size, 76479-006) into a clean scintillation vial. The filtrate was concentrated under reduced pressure and dried under vacuum to afford [Fe(dpa)Cl_2][$FeCl_4$] as a pale orange solid (15.03 mg, 0.026 mmol, 26.4%) give a yellow-orange

solid. Crystals suitable for X-ray diffraction were obtained *via* recrystallization of a concentrated $[Fe(dpa)Cl_2][FeCl_4]$ solution in 1,2-dichloroethane.

Characterization

FTIR–ATR (cm^{-1}): 3210 (m), 2928 (vw) 1607 (s), 1569 (m), 1487 (m), 1438 (s), 1428 (s), 1410 (m), 1308 (w), 1282 (s), 1178 (m). 1157 (m), 1105 (m), 1080 (m), 1026 (m), 997 (m), 944 (m), 815 (m), 762 (s), 722 (m), 672 (m), 643 (m) 505 (w), 471 (m), 429 (m) 414 (m), 322 (sh), 228 (br) 243 (sh)
Elemental Analysis calculated for [C24H32Cl2FeN6O3]: C 27.52%; N 8.02%, H 2.50%; Cl 40.62%; found: C 27.76%, N 7.831%, H 2.49%, Cl 39.92%.

8. General procedure for Fe mediated photodecarboxylative Giese reaction.

A 30.0 µL aliquot of a suspension of *f*ac-Fe(dpa)Cl3 (0.1 M) or stirred mixtures of FeCl3 and dpa (0.1M) in MeOH, MeCN, THF, toluene, DCE, EtOAc, and DMC was dispensed in 1-dram vials (Fisherbrand Class A Glass Vial, 0333925B) and the solvent was evaporated in vacuo with a Genevac EZ-2 4.0 using the machine default “Low BP method”. The reaction vial was equipped with a PTFE-coated stir bar (VWR® spinbar micro, 3 x 10 mm, 58948-375) and was charged with dry acetonitrile (2.00 mL). This was followed by the addition of methionine (24.9 mg, 0.100 mmol, 1.00 equiv.), N,N-diisopropylethyleamine (16.6 µL, 0.100 mmol, 1.00 equiv.), and benzyl acrylate (15.3 µL, 0.100 mmol, 1.00 equiv.). The vial was fitted with a cap with a rubber septum (Chemglass CG-4910-01 and CV-4080-0013 respectively). A 21-gauge needle connected to a positive pressure nitrogen source was submerged in the solution and a vent 21-gauge needle was added to allow the reaction to sparge for 3 min. Both needles were removed from the reaction. The reaction was placed 4 cm away from a 390 nm Kessil lamp and irradiated for 18 h while stirring at 400 rpm. After irradiation was finished, the reaction was filtered through a plug of silica immobilized in a pasteur pipette with glass wool with ethyl acetate, and the filtered crude was concentrated *in vacuo.* Yields were reported as ^{1}H NMR assay yields against dibromomethane (7.01 µL, 0.100 mmol) as an internal standard.

III. UV-Vis spectroscopy.

1. UV-Vis sample preparation.

Each of the $FeCl_3$ and dpa, *fac*-Fe(dpa)Cl_3 controls slurries and the stirred mixtures of $FeCl_3$ and dpa were diluted into a 0.0200 M solution by taking a 0.500 mL aliquot into a 1-dram vial charged with 2.00 ml of a corresponding solvent. UV-Vis spectra were collected in a Hellma quartz microplate (730-009-44, 14.5 x 127 x 85.5 mm). The quartz plate was filled with 200 µL of corresponding solvents and 200 µL of each solvent was dispensed into an empty well as a blank. 4.00 µL of the diluted samples were transferred to an individual well on the quartz plate. The UV-Vis spectra were then collected with the BMG plate reader.

2. UV-Vis spectra

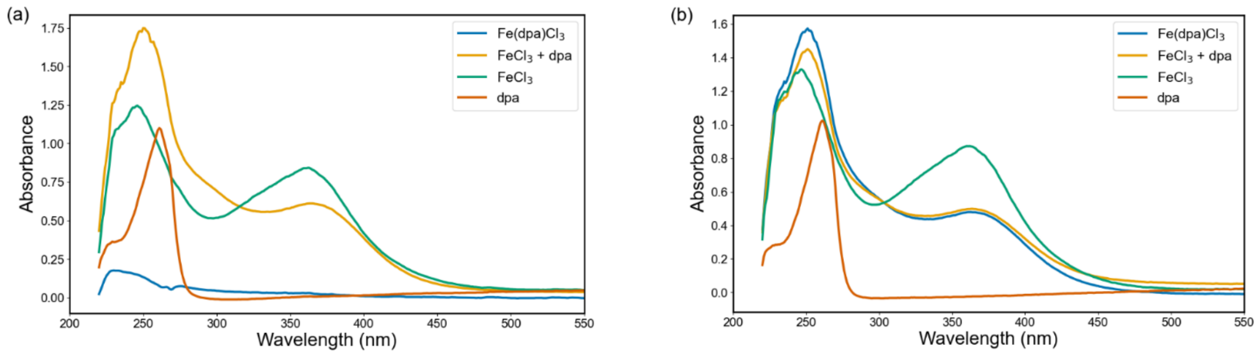

Figure S1. Evaluation of $FeCl_3$ and dpa stirred solution via UV-Vis in MeOH. (a) UV-Vis spectrum of $FeCl_3$ and dpa stirred solution in MeOH at 25 °C. (b) UV-Vis spectrum of $FeCl_3$ and dpa stirred solution in MeOH at 60 °C.

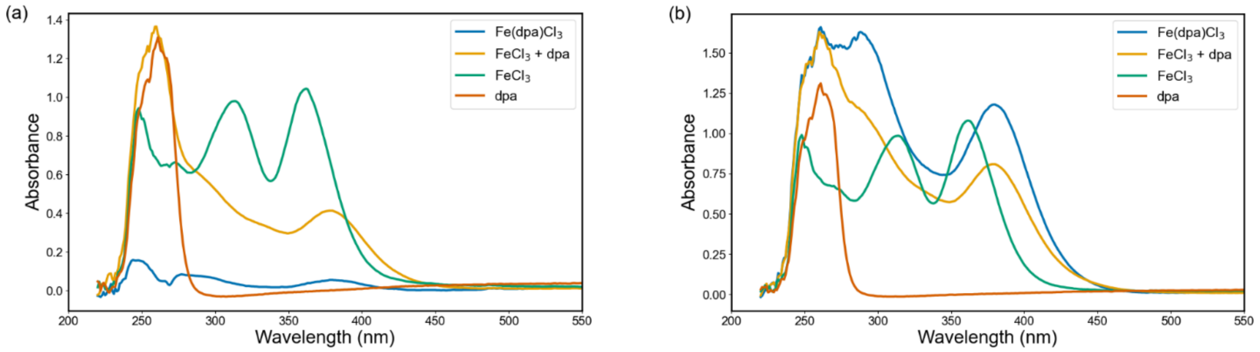

Figure S2. Evaluation of $FeCl_3$ and dpa stirred solution via UV-Vis in MeCN. (a) UV-Vis spectrum of $FeCl_3$ and dpa stirred solution in MeCN at 25 °C. (b) UV-Vis spectrum of $FeCl_3$ and dpa stirred solution in MeCN at 60 °C.

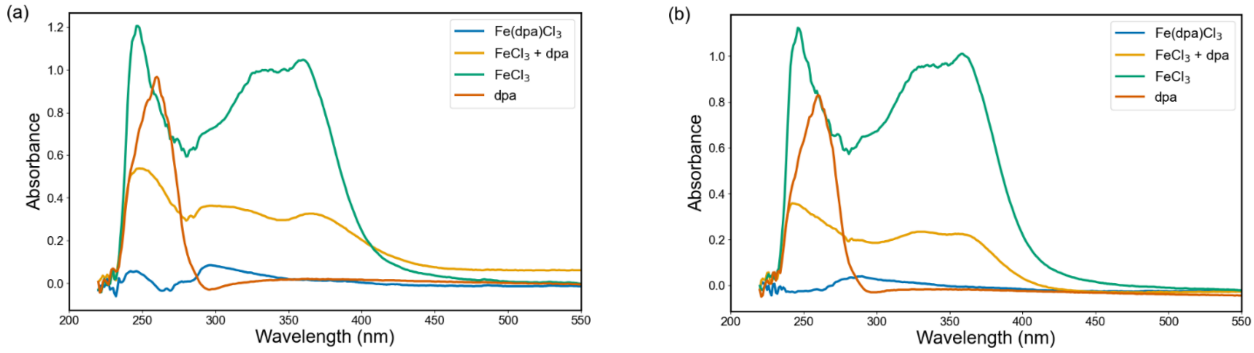

Figure S3. Evaluation of $FeCl_3$ and dpa stirred solution via UV-Vis in THF. (a) UV-Vis spectrum of $FeCl_3$ and dpa stirred solution in THF at 25 °C. (b) UV-Vis spectrum of $FeCl_3$ and dpa stirred solution in THF at 60 °C.

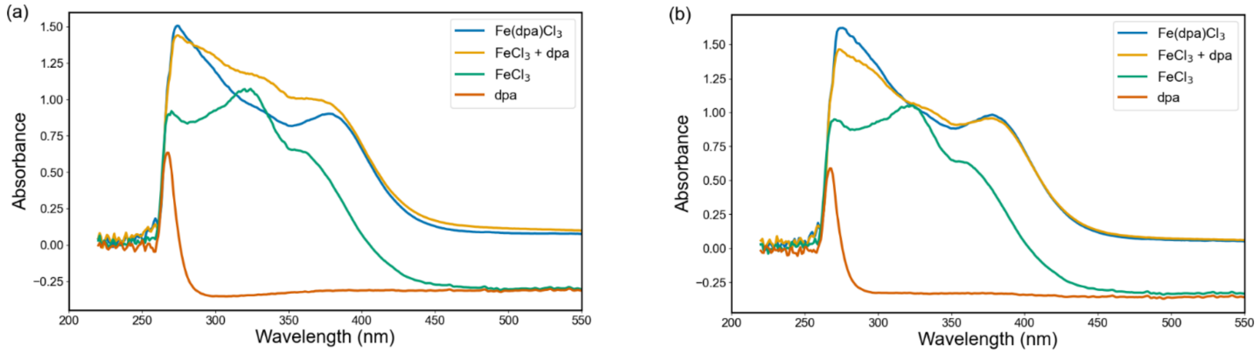

Figure S4. Evaluation of $FeCl_3$ and dpa stirred solution via UV-Vis in toluene. (a) UV-Vis spectrum of $FeCl_3$ and dpa stirred solution in toluene at 25 °C. (b) UV-Vis spectrum of $FeCl_3$ and dpa stirred solution in toluene at 60 °C.

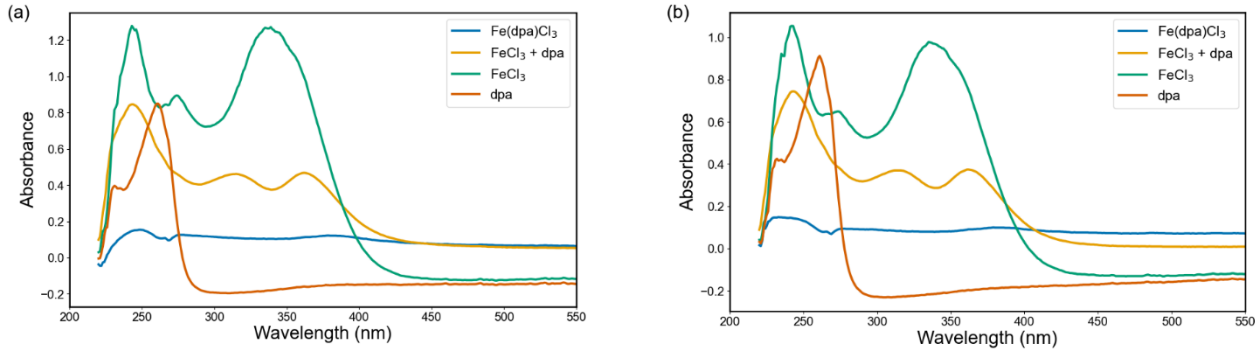

Figure S5. Evaluation of $FeCl_3$ and dpa stirred solution via UV-Vis in DCE. (a) UV-Vis spectrum of $FeCl_3$ and dpa stirred solution in DCE at 25 °C. (b) UV-Vis spectrum of $FeCl_3$ and dpa stirred solution in DCE at 60 °C.

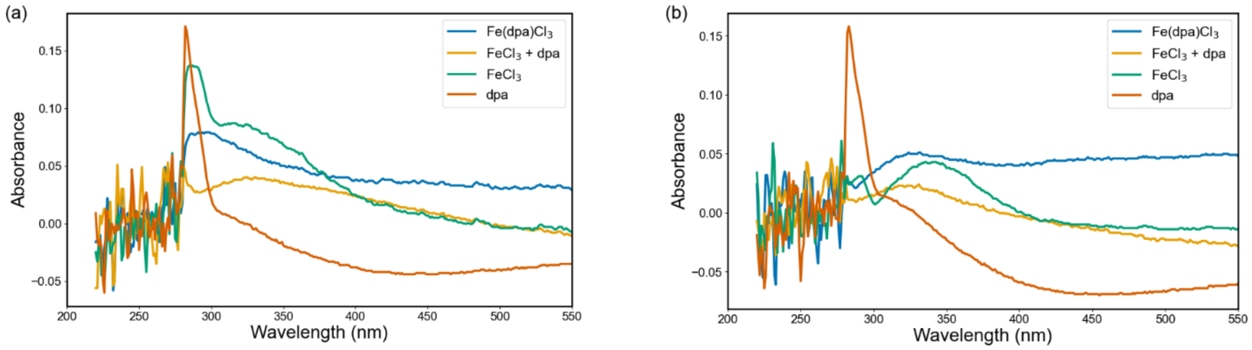

Figure S6. Evaluation of $FeCl_3$ and dpa stirred solution via UV-Vis in EtOAc. (a) UV-Vis spectrum of $FeCl_3$ and dpa stirred solution in EtOAc at 25 °C. (b) UV-Vis spectrum of $FeCl_3$ and dpa stirred solution in EtOAc at 60 °C.

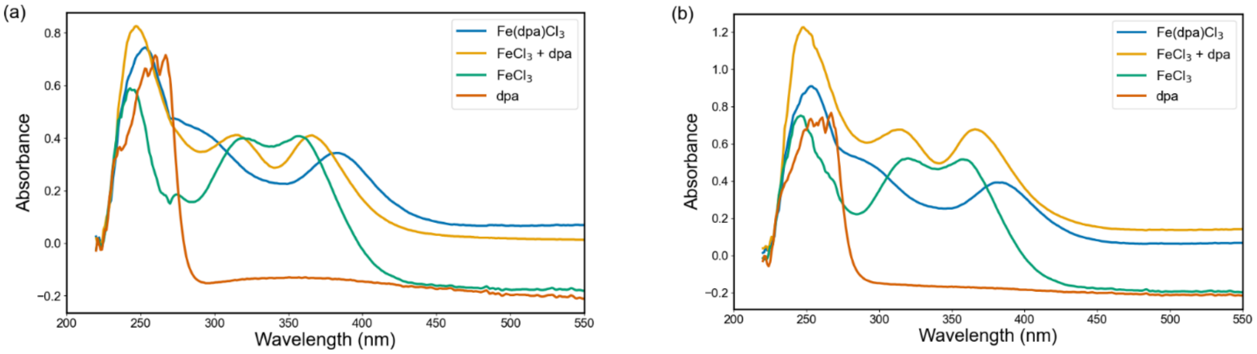

Figure S7. Evaluation of $FeCl_3$ and dpa stirred solution via UV-Vis in DMC. (a) UV-Vis spectrum of $FeCl_3$ and dpa stirred solution in DMC at 25 °C. (b) UV-Vis spectrum of $FeCl_3$ and dpa stirred solution in DMC at 60 °C.

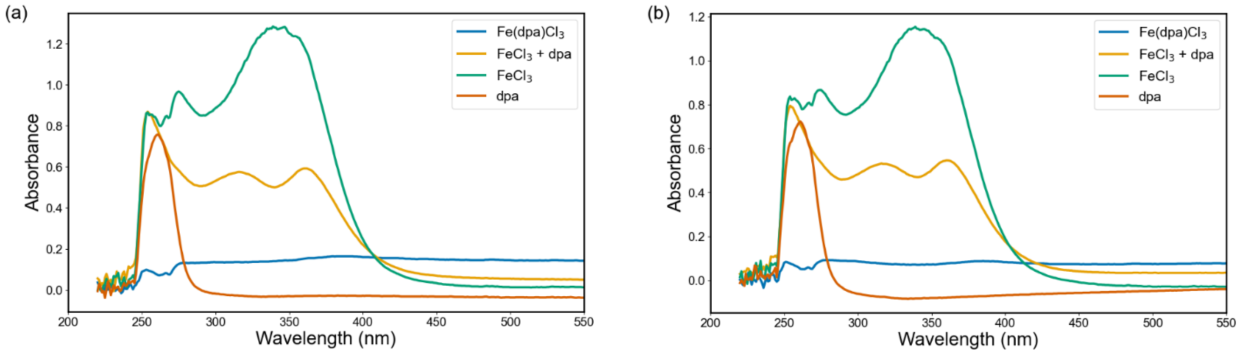

Figure S8. Evaluation of $FeCl_3$ and dpa stirred solution via UV-Vis in DMF. (a) UV-Vis spectrum of $FeCl_3$ and dpa stirred solution in DMF at 25 °C. (b) UV-Vis spectrum of $FeCl_3$ and dpa stirred solution in DMF at 60 °C.

IV. TGA analysis.

1. TGA procedure.

Reproducibility study of TGA replicates for samples of Fe(dpa)Cl_3

Fe(dpa)Cl_3 (1.81 mg, 0.0050 mmol) was placed on three 70 µL platinum crucible (Mettler Toledo, 51119654) and these three samples were analyzed by TGA under an inert atmosphere (nitrogen). TGA was performed under constant N_2 flow (50 mL/min). After sample insertion, the sample method had a 10-minute hold time at 25 °C to flush the furnace with N_2 and was further heated to 650 °C at a ramp rate of 10 °C/min. The sample weight was normalized to 100 wt. %. The procedure was repeated an additional time using a different sample of Fe(dpa)Cl_3.

Analysis for fac-Fe(dpa)Cl_3, $FeCl_3$, dpa, [Fe(dpa)$_2$]Cl_2[2CH_3OH], [Fe(dpa)Cl_2][$FeCl_4$] and solids produced from stirring $FeCl_3$ and dpa

Solids (0.0050 mmol) were placed on 70 µL platinum crucible (Mettler Toledo, 51119654) and analyzed by TGA under an inert atmosphere (nitrogen). TGA was performed under constant N_2 flow (50 mL/min). After sample insertion, the sample method had a 10-minute hold time at 25 °C to flush the furnace with N_2 and was further heated to 650 °C at a ramp rate of 10 °C/min. The sample weight was normalized to 100 wt. %.

Analysis for fac-Fe(dpa)Cl_3 and stirred mixtures of $FeCl_3$ and dpa in MeOH, MeCN, THF, toluene, DCE, EtOAc, and DMC

A 50.0 µL aliquot of reaction crude was dispensed into 70 µL platinum crucible (Mettler Toledo, 51119654) while the solution was stirred. TGA was performed under constant N_2 flow (50 mL/min). After the sample insertion, the method had a 10-minute hold at 25 °C to flush the furnace with N_2 and heated to 82 °C at a ramp rate of 10 °C/min, held at 82 °C for 10 minutes, and then heated to 650 °C at a ramp rate of 10 °C/min. The sample weight was normalized to 100 wt. % after solvent removal during the second isothermal hold at 82 °C.

Analysis for fac-Fe(dpa)Cl_3 and stirred mixture of $FeCl_3$ and dpa in DMF

A 50.0 µL aliquot of reaction crude was dispensed into 70 µL platinum crucible (Mettler Toledo, 51119654) while the solution was stirred. TGA was performed under constant N_2 flow (50 mL/min). After sample insertion, the method had a 10-minute hold at 25 °C to flush the furnace with N_2 and heated to 82 °C at a ramp rate of 10 °C/min, held at 82 °C for 35 minutes, and then heated to 650 °C at a ramp rate of 10 °C/min. The sample weight was normalized to 100 wt. % after solvent removal during the second isothermal hold at 82 °C.

Analysis for mixtures of fac-Fe(dpa)Cl_3 and [Fe(dpa)$_2$]Cl_2 [2CH_3OH]

A mixture of both solids in different ratios based on Table S1 were placed on 70 µL platinum crucible (Mettler Toledo, 51119654) and analyzed by TGA under an inert atmosphere

(nitrogen). TGA was performed under constant N_2 flow (50 mL/min). After sample insertion, the sample method had a 10-minute hold time at 25 °C to flush the furnace with N_2 and was further heated to 650 °C at a ramp rate of 10 °C/min. The sample weight was normalized to 100 wt. %.

Table S1. Summary of analysis for mixture samples of *fac*-Fe(dpa)Cl_3 and $[Fe(dpa)_2]Cl_2[2CH_3OH]$.

$[Fe(dpa)_2]Cl_2[2CH_3OH]$ (mg)	Fe(dpa)Cl_3 (mg)	$[Fe(dpa)_2]Cl_2[2CH_3OH]$: Fe(dpa)Cl_3
4	0	1:0
2	2	1:1
0	4	0:1

2. Reproducibility studies of TGA for *fac*-Fe(dpa)Cl_3 samples

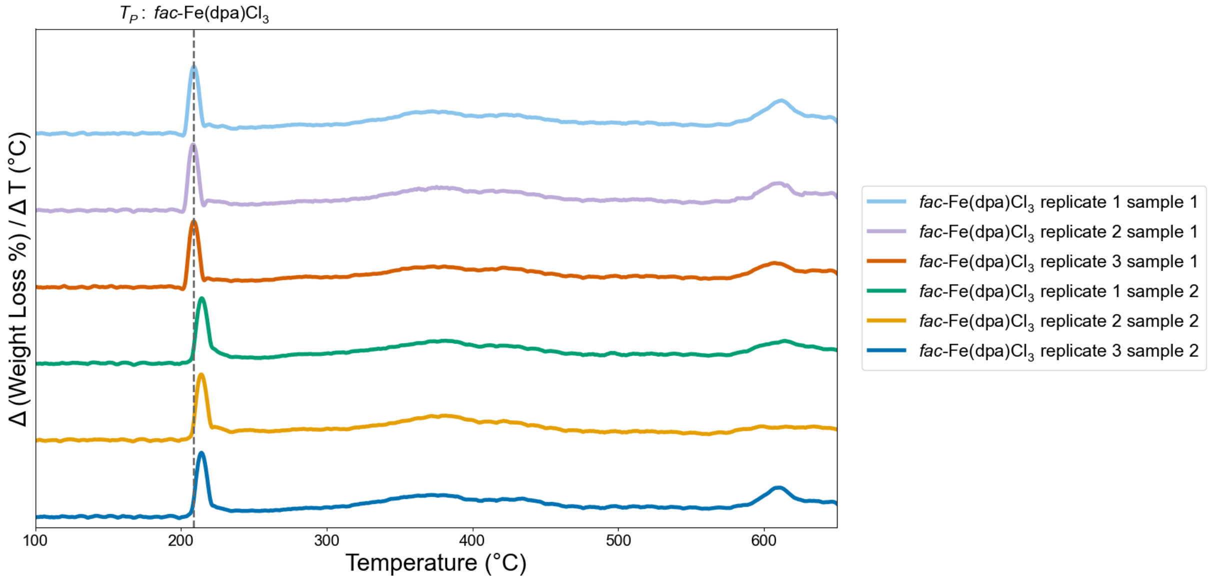

Figure S9. Triplicate TGA plots of *fac*-Fe(dpa)Cl_3 across 2 different samples.

3. TGA Analysis of mixtures of *fac*-Fe(dpa)Cl_3 and [Fe(dpa)$_2$]Cl_2[2CH_3OH]

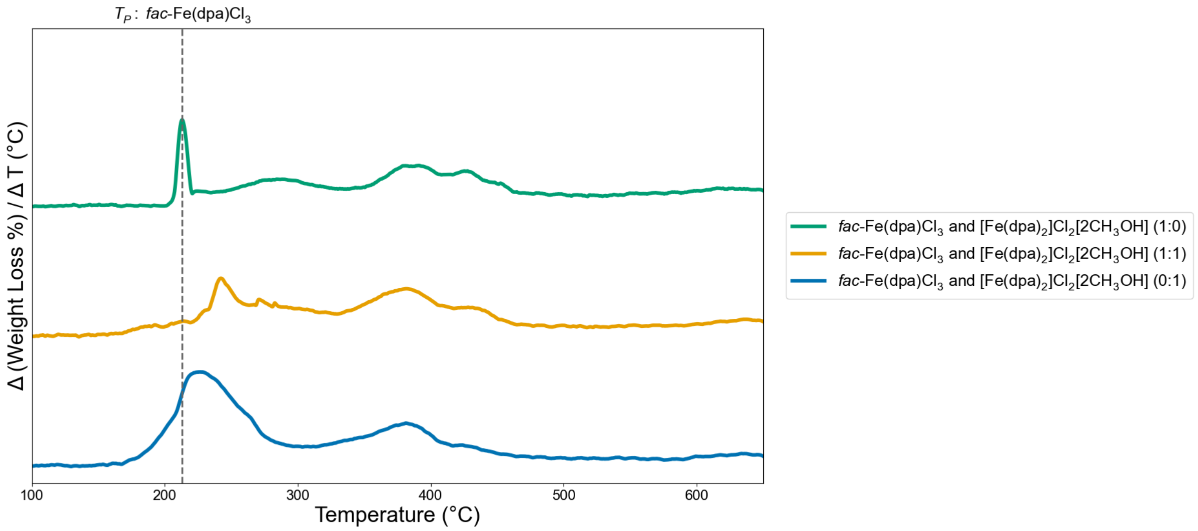

Figure S10. TGA analysis of *fac*-Fe(dpa)Cl_3 and [Fe(dpa)$_2$]Cl_2[2CH_3OH] in different weight ratios from 0:1, 1:1, and 1:0.

4. Overlaid first derivative TGA of Fe(dpa)Cl_3 and mixtures of $FeCl_3$ and dpa in various solvents.

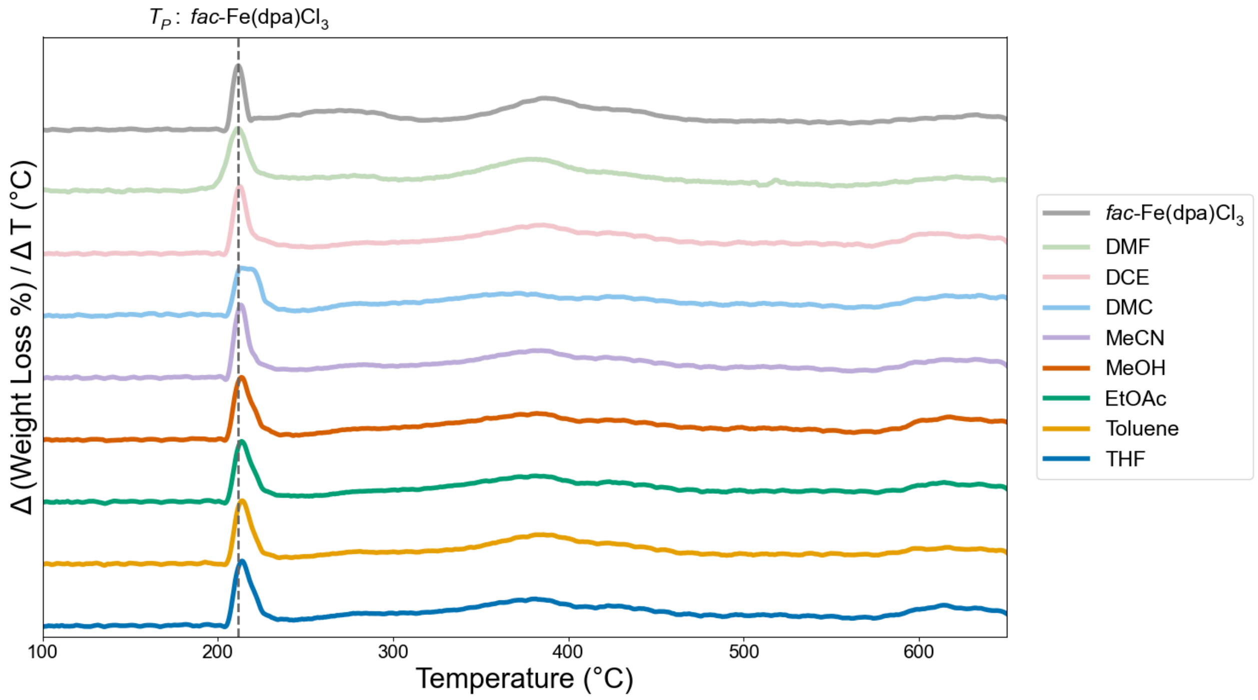

Figure S11. First derivative TGA of Fe(dpa)Cl_3 stirred in various solvents at 25 °C, ordered by ascending T_p.

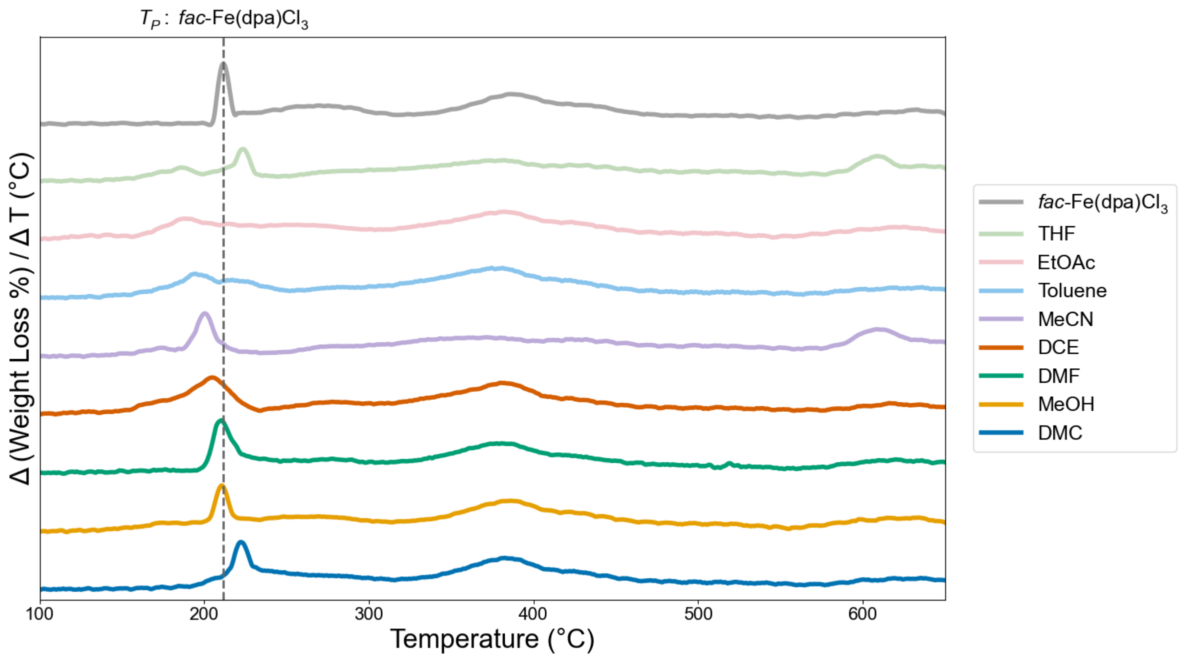

Figure S12. First derivative TGA of $FeCl_3$ and dpa stirred in various solvents at 25 °C ordered by ascending T_p.

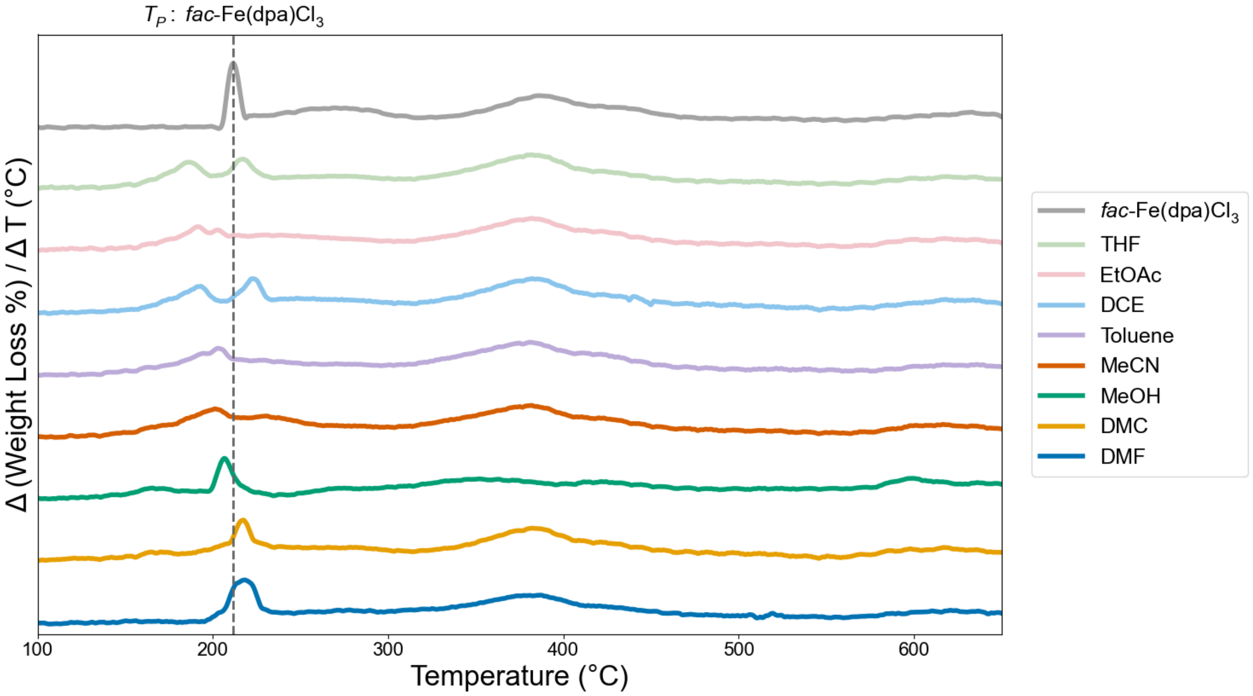

Figure S13. First derivative TGA of $FeCl_3$ and dpa stirred at 60 °C ordered by ascending T_p.

5. TGA Profiles of $FeCl_3$ and dpa solutions at 25 °C.

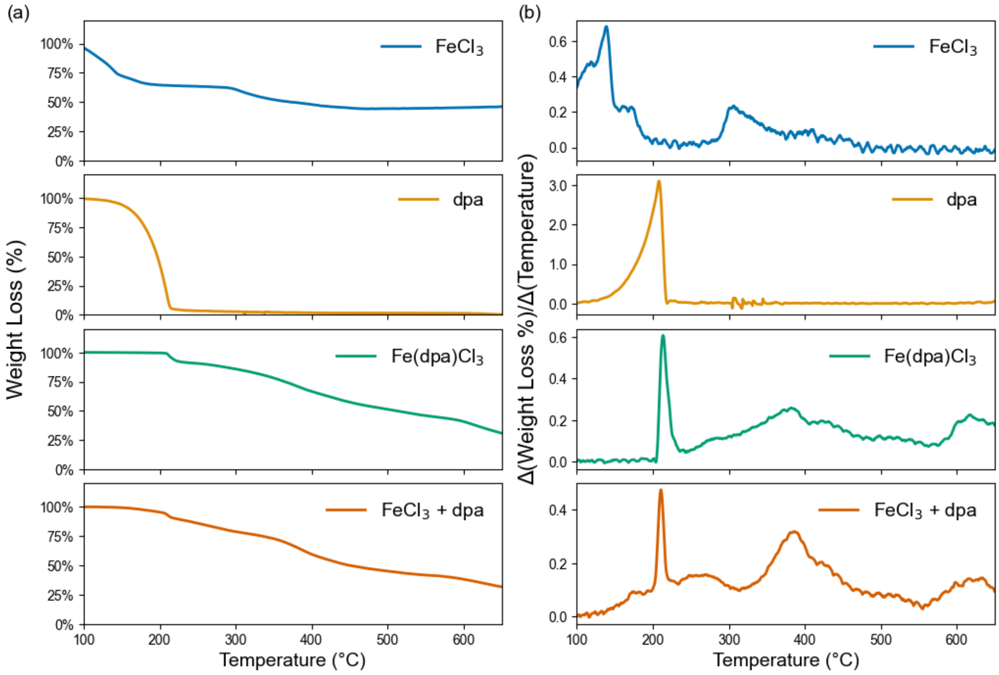

Figure S14. Evaluation of $FeCl_3$ and dpa solution via TGA in MeOH. (a) TGA profile for $FeCl_3$ and dpa in MeOH. "Fe(dpa)Cl_3" refers to the pure complex stirred in the corresponding solvent at 25 °C. "$FeCl_3$ + dpa" refers to the $FeCl_3$ and dpa mixture solution stirred in the corresponding solvent at 25 °C. (b) First derivative TGA profiles for the corresponding samples.

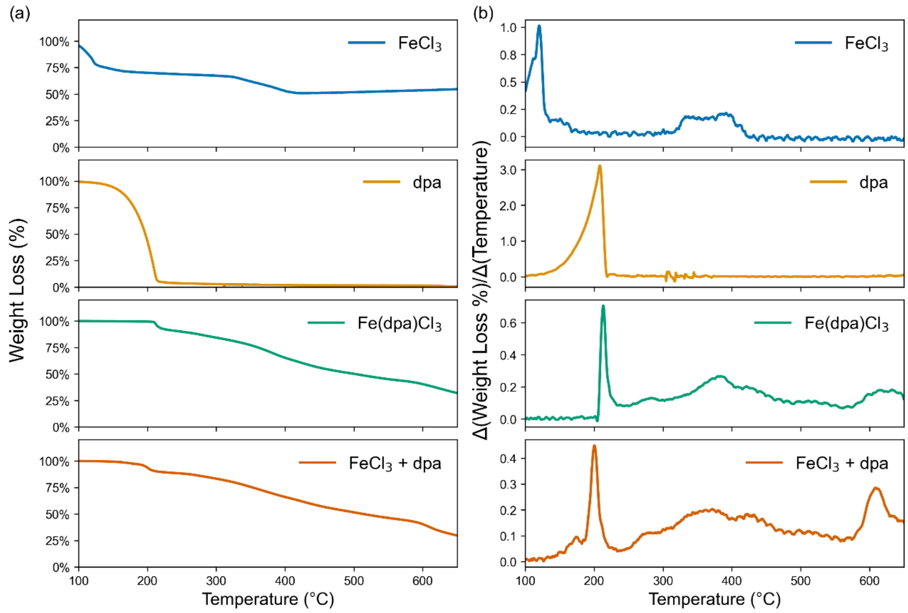

Figure S15. Evaluation of $FeCl_3$ and dpa solution via TGA in MeCN. (a) TGA profile for $FeCl_3$ and dpa in MeCN. "Fe(dpa)Cl_3" refers to the pure complex stirred in the corresponding solvent at 25 °C. "$FeCl_3$ + dpa" refers to the $FeCl_3$ and dpa mixture solution stirred in the corresponding solvent at 25 °C. (b) First derivative TGA profiles for the corresponding samples.

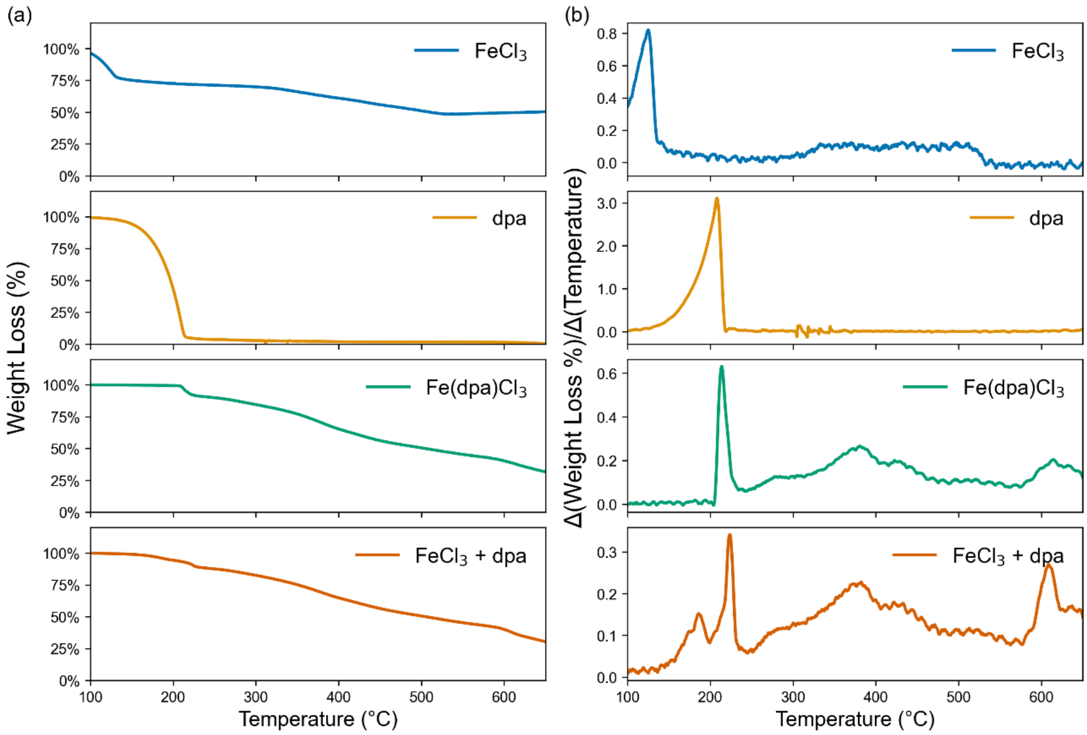

Figure S16. Evaluation of $FeCl_3$ and dpa solution via TGA in THF. (a) TGA profile for $FeCl_3$ and dpa in THF. "Fe(dpa)Cl_3" refers to the pure complex stirred in the corresponding solvent at 25 °C. "$FeCl_3$ + dpa" refers to the $FeCl_3$ and dpa mixture solution stirred in the corresponding solvent at 25 °C. (b) First derivative TGA profiles for the corresponding samples.

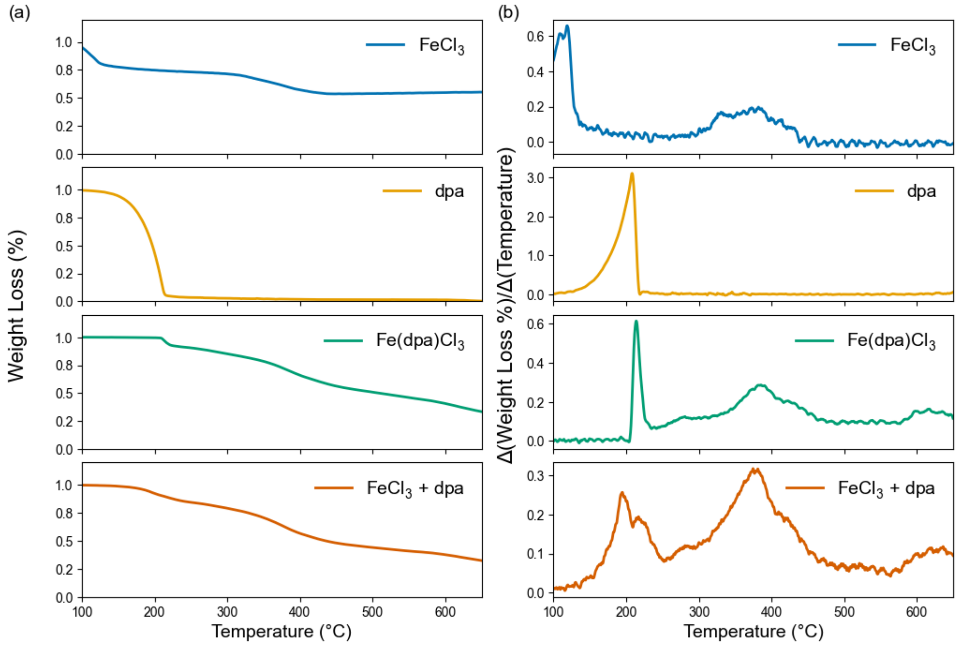

Figure S17. Evaluation of $FeCl_3$ and dpa solution via TGA in toluene. (a) TGA profile for $FeCl_3$ and dpa in toluene. "Fe(dpa)Cl_3" refers to the pure complex stirred in the corresponding solvent at 25 °C. "$FeCl_3$ + dpa" refers to the $FeCl_3$ and dpa mixture solution stirred in the

corresponding solvent at 25 °C. (b) First derivative TGA profiles for the corresponding samples.

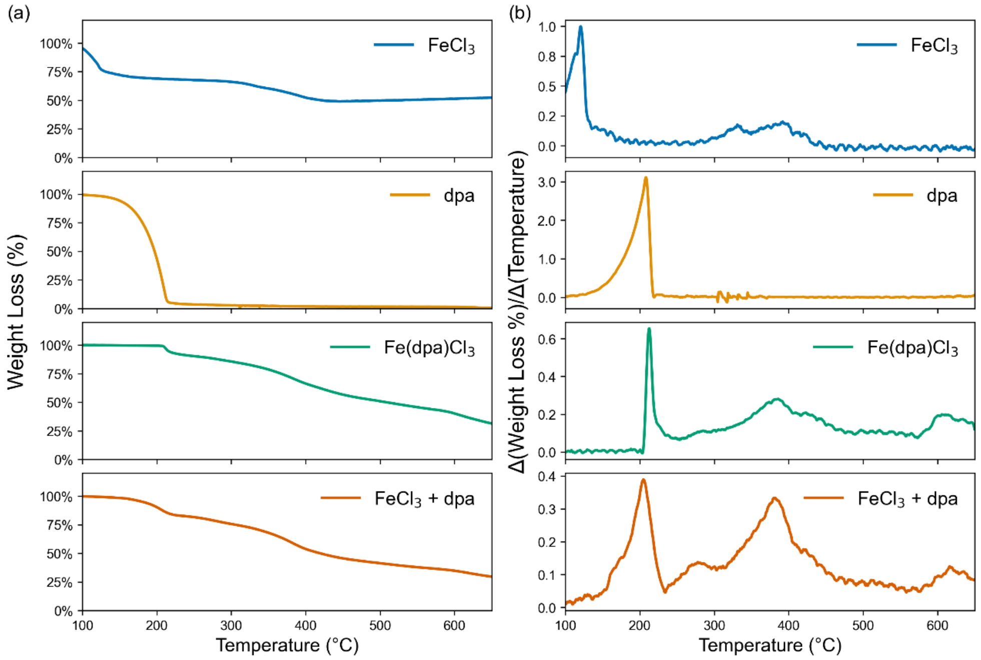

Figure S18. Evaluation of $FeCl_3$ and dpa solution via TGA in DCE. (a) TGA profile for $FeCl_3$ and dpa in DCE. "Fe(dpa)Cl_3" refers to the pure complex stirred in the corresponding solvent at 25 °C. "$FeCl_3$ + dpa" refers to the $FeCl_3$ and dpa mixture solution stirred in the corresponding solvent at 25 °C. (b) First derivative TGA profiles for the corresponding samples.

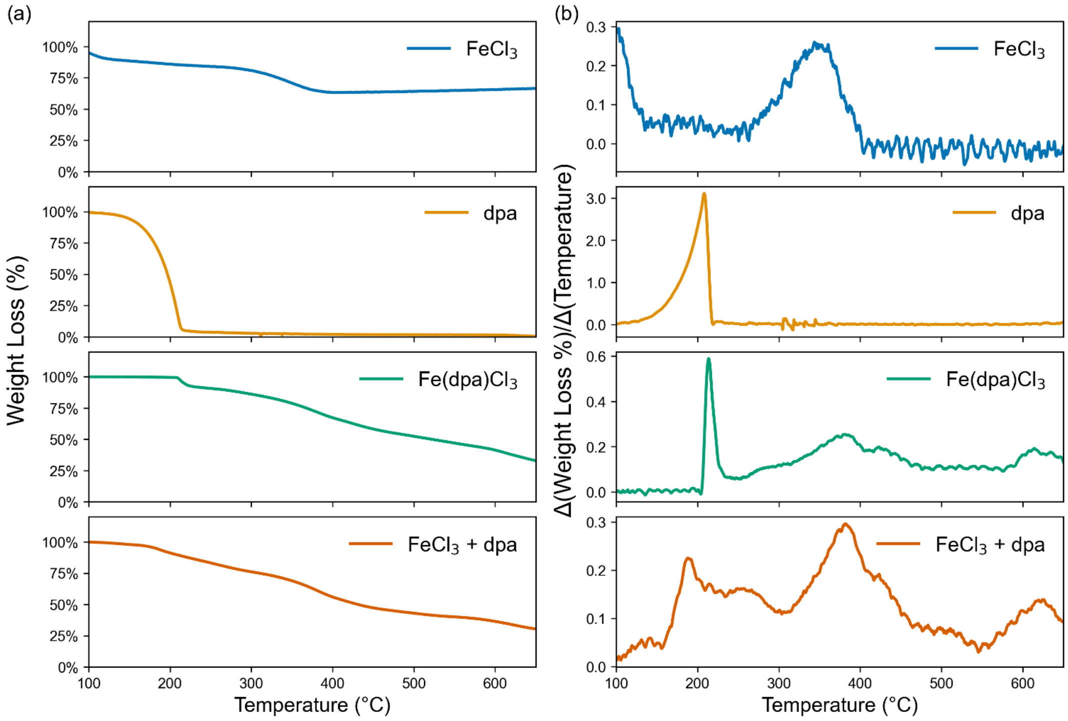

Figure S19. Evaluation of $FeCl_3$ and dpa solution via TGA in EtOAc. (a) TGA profile for $FeCl_3$ and dpa in EtOAc. "Fe(dpa)Cl_3" refers to the pure complex stirred in the corresponding

solvent at 25 °C. "$FeCl_3$ + dpa" refers to the $FeCl_3$ and dpa mixture solution stirred in the corresponding solvent at 25 °C. (b) First derivative TGA profiles for the corresponding samples.

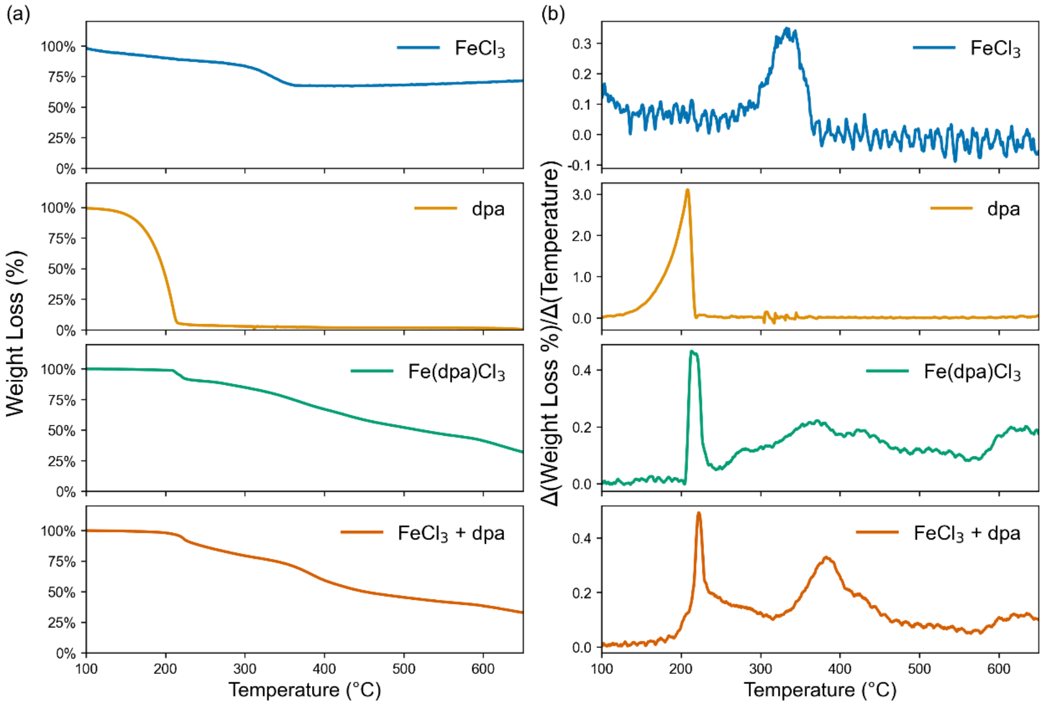

Figure S20. Evaluation of $FeCl_3$ and dpa solution via TGA in DMC. (a) TGA profile for $FeCl_3$ and dpa in DMC. "Fe(dpa)Cl_3" refers to the pure complex stirred in the corresponding solvent at 25 °C. "$FeCl_3$ + dpa" refers to the $FeCl_3$ and dpa mixture solution stirred in the corresponding solvent at 25 °C. (b) First derivative TGA profiles for the corresponding samples.

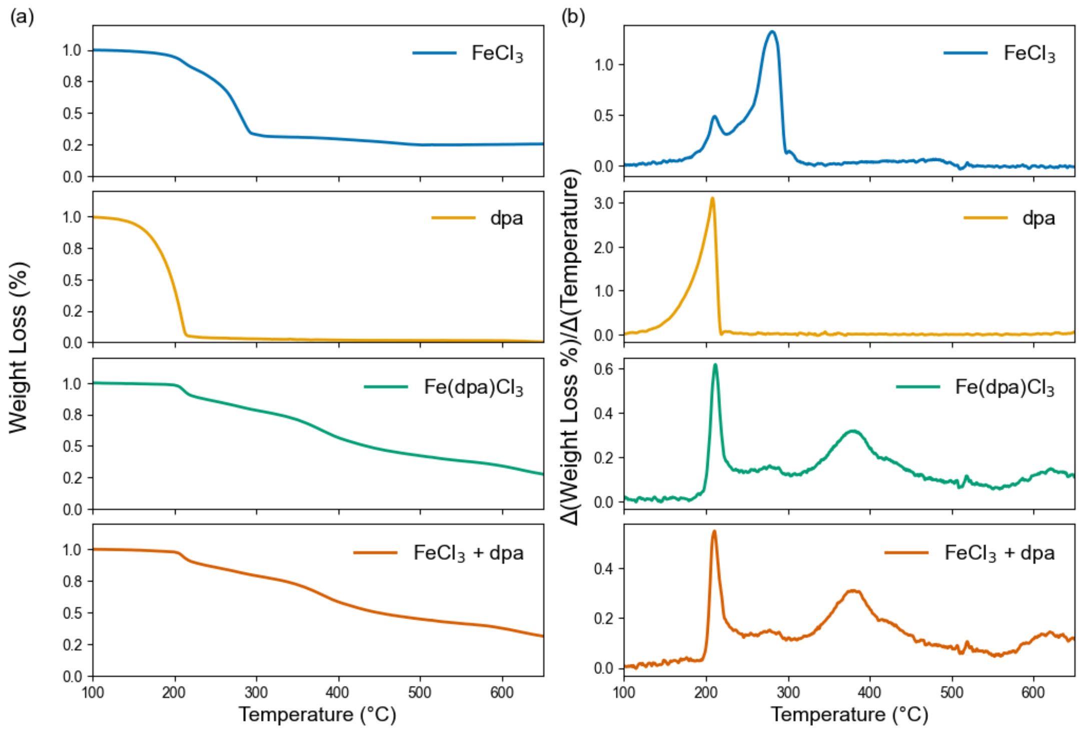

Figure S21. Evaluation of $FeCl_3$ and dpa solution via TGA in DMF. (a) TGA profile for $FeCl_3$ and dpa in DMF. “Fe(dpa)Cl_3” refers to the pure complex stirred in the corresponding solvent at 25 °C. “$FeCl_3$ + dpa” refers to the $FeCl_3$ and dpa mixture solution stirred in the corresponding solvent at 25 °C. (b) First derivative TGA profiles for the corresponding samples.

6. TGA Profiles of $FeCl_3$ and dpa solutions at 60 °C.

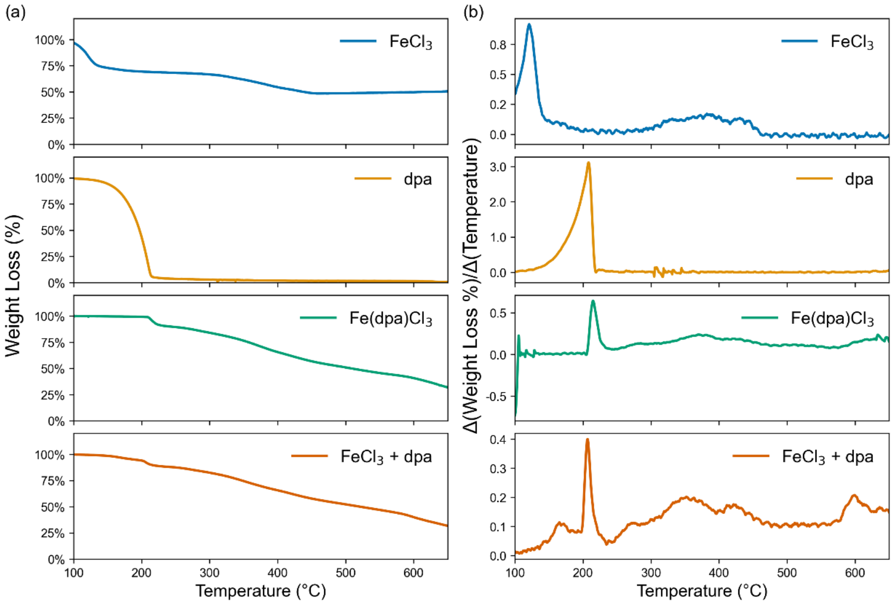

Figure S22. Evaluation of $FeCl_3$ and dpa solution via TGA in MeOH. (a) TGA profile for $FeCl_3$ and dpa in MeOH. “Fe(dpa)Cl_3” refers to the pure complex stirred in the corresponding solvent at 60 °C. “$FeCl_3$ + dpa” refers to the $FeCl_3$ and dpa mixture solution stirred in the corresponding solvent at 60°C. (b) First derivative TGA profiles for the corresponding samples.

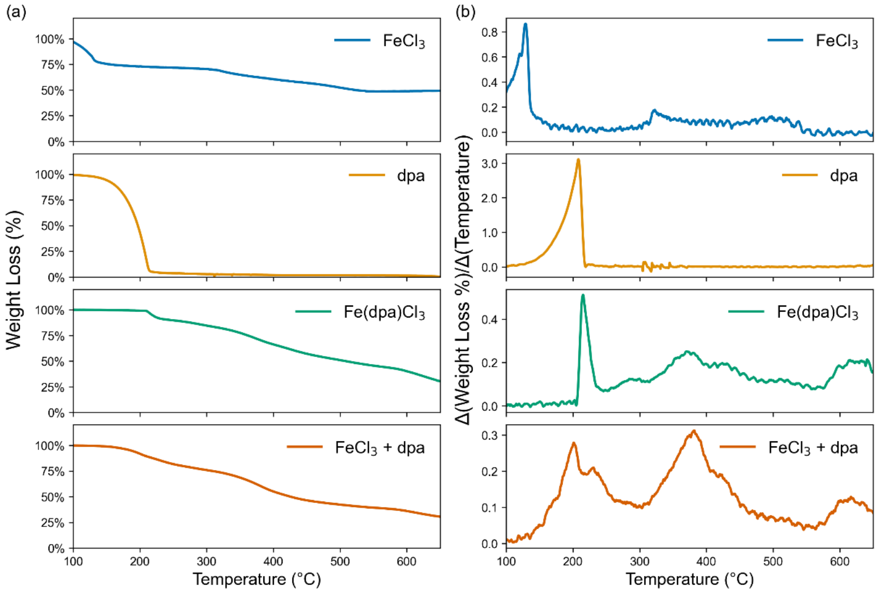

Figure S23. Evaluation of $FeCl_3$ and dpa solution via TGA in MeCN. (a) TGA profile for $FeCl_3$ and dpa in MeCN. “Fe(dpa)Cl_3” refers to the pure complex stirred in the corresponding

solvent at 60 °C. "$FeCl_3$ + dpa" refers to the $FeCl_3$ and dpa mixture solution stirred in the corresponding solvent at 60°C. (b) First derivative TGA profiles for the corresponding samples.

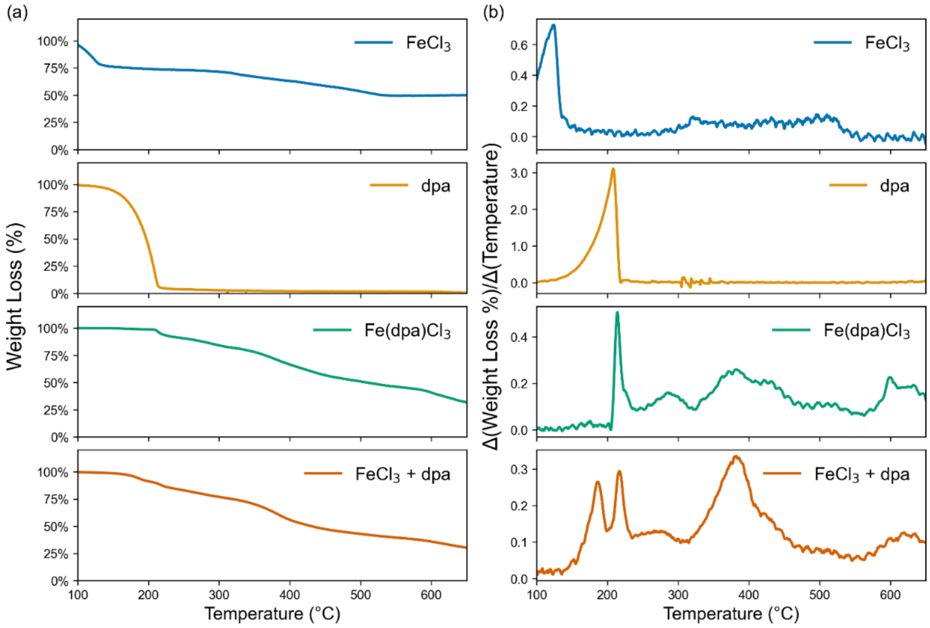

Figure S24. Evaluation of $FeCl_3$ and dpa solution via TGA in THF. (a) TGA profile for $FeCl_3$ and dpa in THF. "Fe(dpa)Cl_3" refers to the pure complex stirred in the corresponding solvent at 60 °C. "$FeCl_3$ + dpa" refers to the $FeCl_3$ and dpa mixture solution stirred in the corresponding solvent at 60°C. (b) First derivative TGA profiles for the corresponding samples.

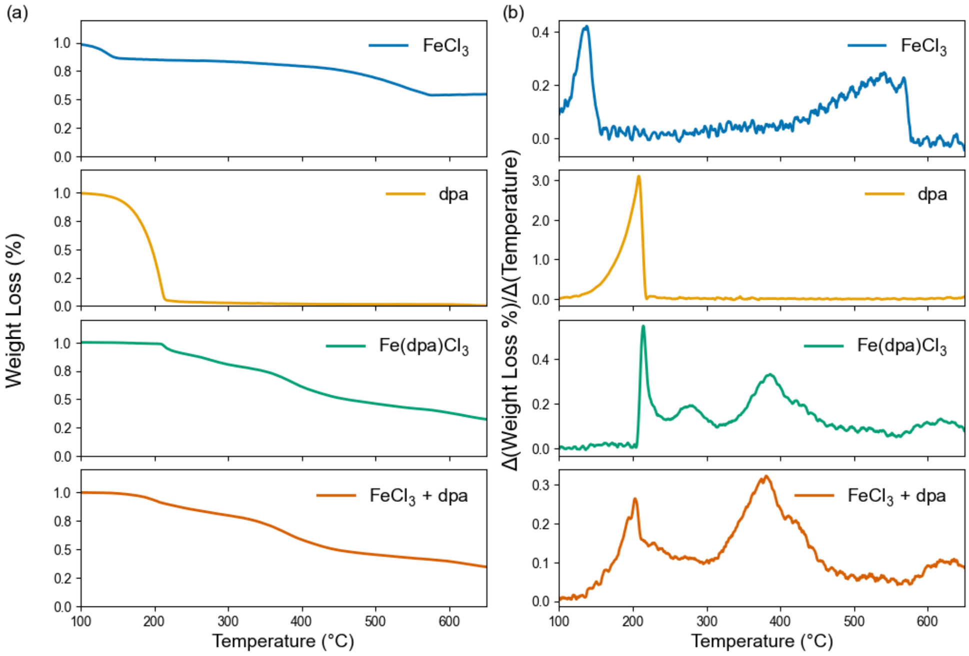

Figure S25. Evaluation of $FeCl_3$ and dpa solution via TGA in toluene. (a) TGA profile for $FeCl_3$ and dpa in toluene. "Fe(dpa)Cl_3" refers to the pure complex stirred in the corresponding solvent at 60 °C. "$FeCl_3$ + dpa" refers to the $FeCl_3$ and dpa mixture solution stirred in the

corresponding solvent at 60°C. (b) First derivative TGA profiles for the corresponding samples.

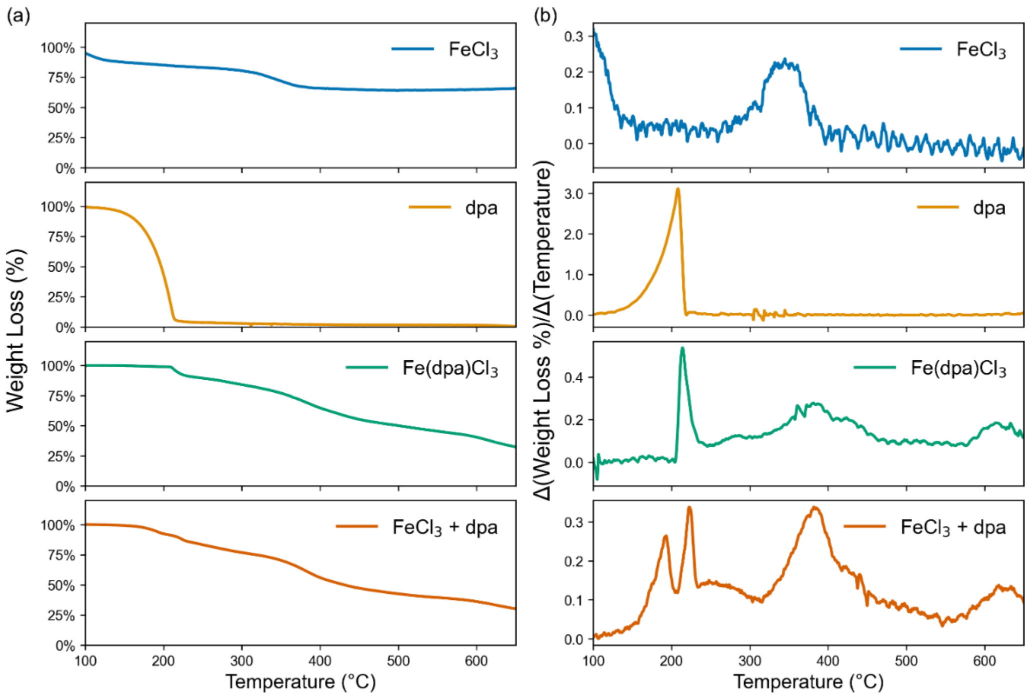

Figure S26. Evaluation of $FeCl_3$ and dpa solution via TGA in DCE. (a) TGA profile for $FeCl_3$ and dpa in DCE. "Fe(dpa)Cl_3" refers to the pure complex stirred in the corresponding solvent at 60 °C. "$FeCl_3$ + dpa" refers to the $FeCl_3$ and dpa mixture solution stirred in the corresponding solvent at 60°C. (b) First derivative TGA profile for the corresponding samples.

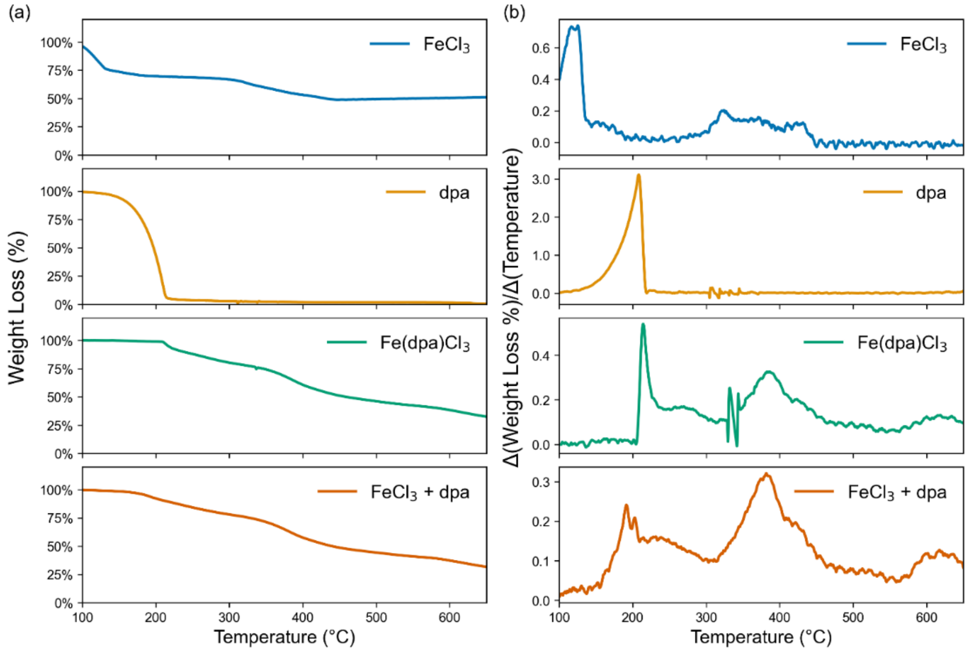

Figure S27. Evaluation of $FeCl_3$ and dpa solution via TGA in EtOAc. (a) TGA profile for $FeCl_3$ and dpa in EtOAc. "Fe(dpa)Cl_3" refers to the pure complex stirred in the corresponding solvent at 60 °C. "$FeCl_3$ + dpa" refers to the $FeCl_3$ and dpa mixture solution stirred in the

corresponding solvent at 60°C. (b) First derivative TGA profiles for the corresponding samples.

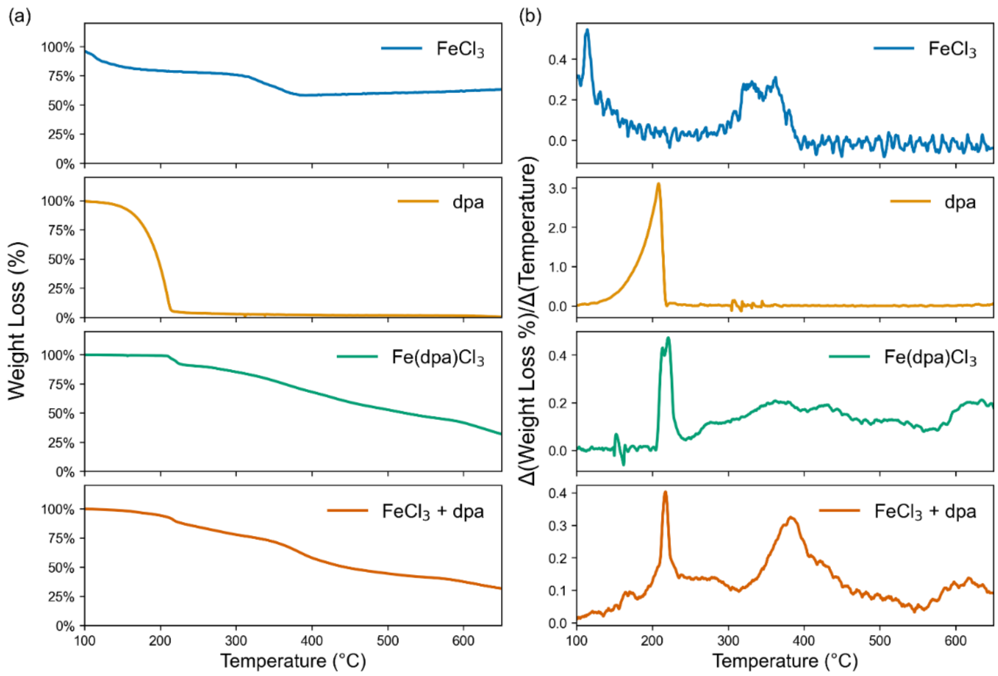

Figure S28. Evaluation of $FeCl_3$ and dpa solution via TGA in DMC. (a) TGA profile for $FeCl_3$ and dpa in DMC. "Fe(dpa)Cl_3" refers to the pure complex stirred in the corresponding solvent at 60 °C. "$FeCl_3$ + dpa" refers to the $FeCl_3$ and dpa mixture solution stirred in the corresponding solvent at 60°C. (b) First derivative TGA profiles for the corresponding samples.

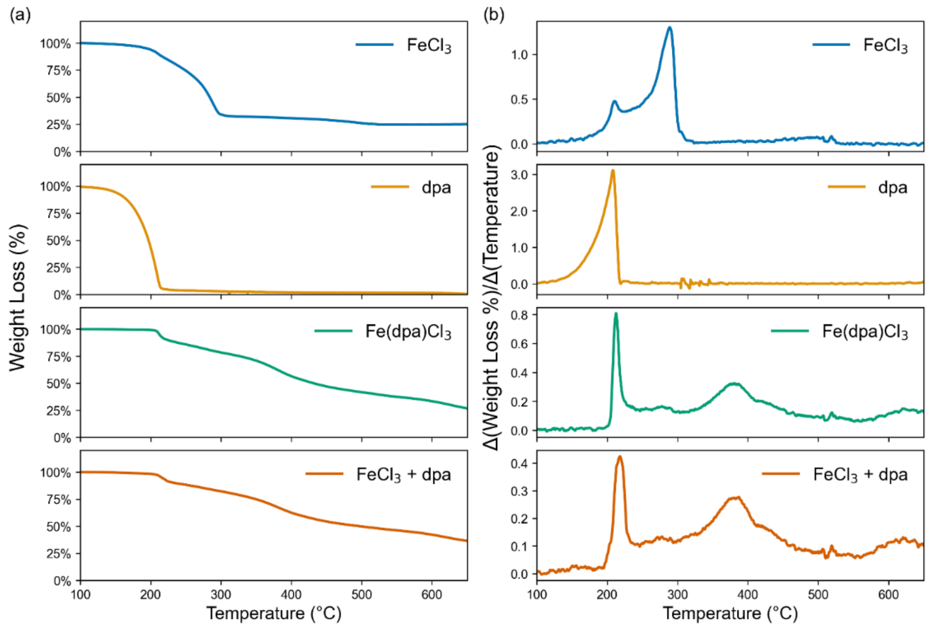

Figure S29. Evaluation of $FeCl_3$ and dpa solution via TGA in DMF. (a) TGA profile for $FeCl_3$ and dpa in DMF. "Fe(dpa)Cl_3" refers to the pure complex stirred in the corresponding solvent at 60 °C. "$FeCl_3$ + dpa" refers to the $FeCl_3$ and dpa mixture solution stirred in the

corresponding solvent at 60°C. (b) First derivative TGA profiles for the corresponding samples.

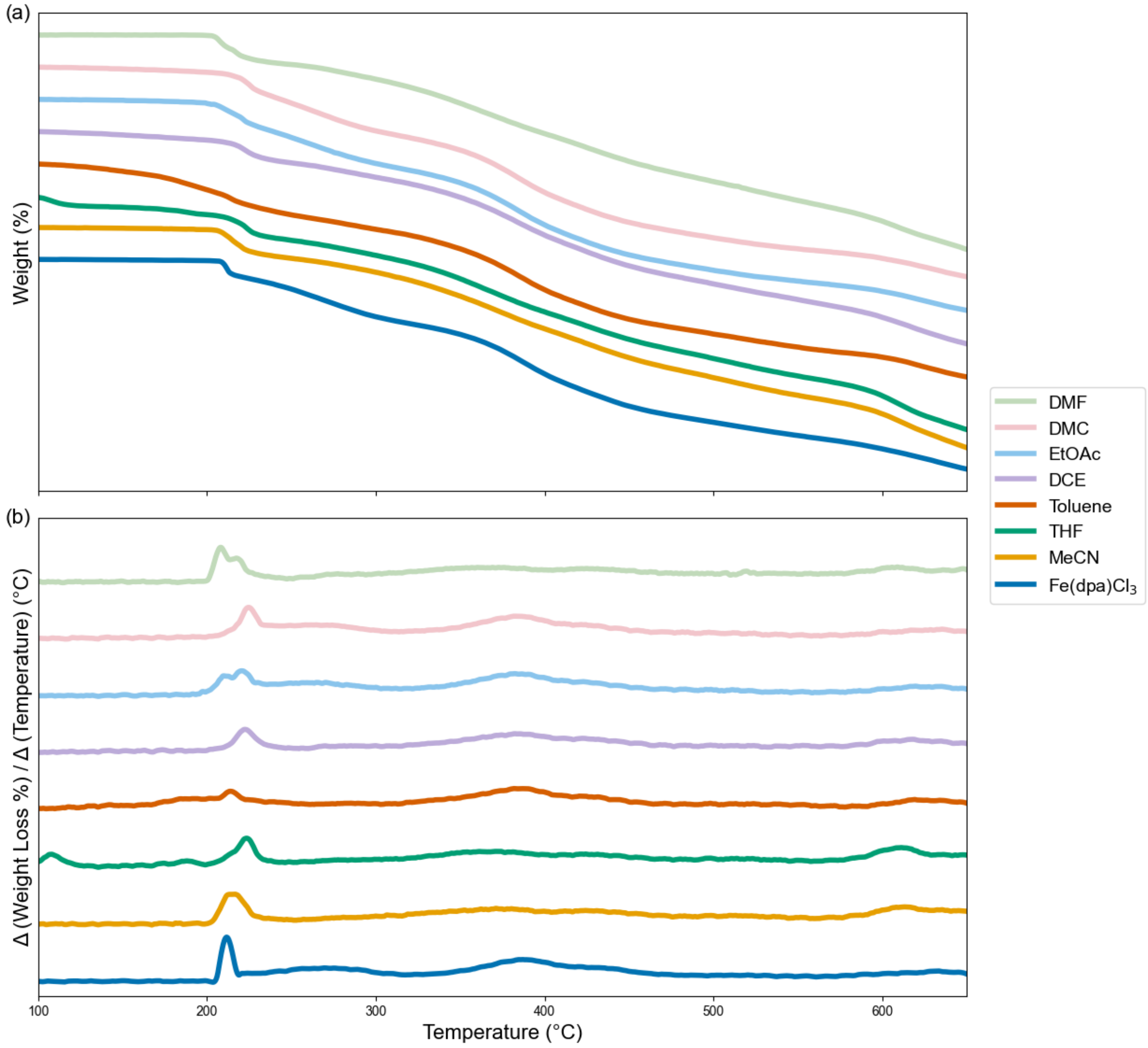

Figure S30. (a) TGA profiles of solids obtained from IR spectra produced from stirring $FeCl_3$ and dpa in MeOH, MeCN, THF, toluene, DCE, EtOAc, DMC, and DMF at 25 °C and (b) first derivative of TGA profiles of solids obtained from IR spectra produced from stirring $FeCl_3$ and dpa in MeOH, MeCN, THF, toluene, DCE, EtOAc, DMC, and DMF at 25 °C.

V. Characterization of *fac*-Fe(dpa)Cl_3 Degradation through Gas Chromatography Mass Spectrometry (GCMS).

30.0 mg of Fe(dpa)Cl_3 was added to a 50.0 mL pressure vessel tube sealed in glove box. The tube was brought outside, placed in a 20 mL scintillation vial aluminum reaction block (Chemglass, CG-1991-01) and heated to 230 °C for 1 hour. The tube was then allowed to cool to room temperature. Liquid on the wall of the tube as a result from condensation during the reaction was rinsed down with ethyl acetate and analyzed via GCMS. The sample showed one major peak at 2.111 min, which has a MS fragmentation consistent with 2-methylpyridine.

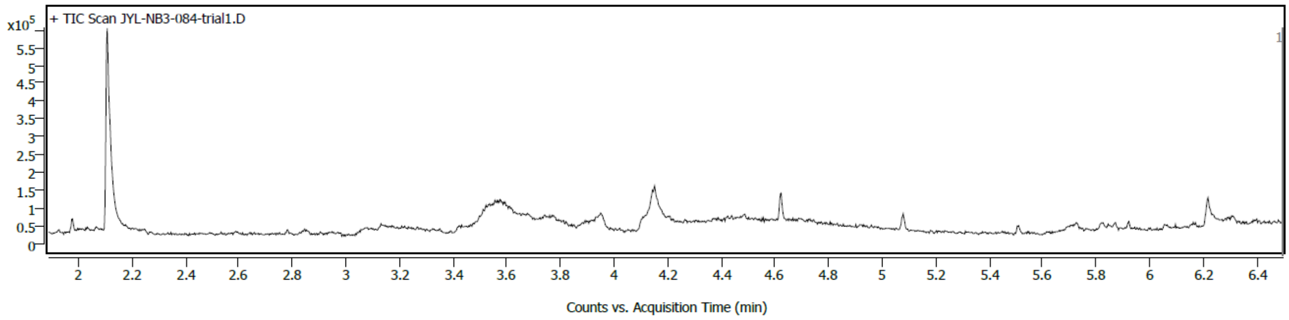

Figure S31. GCMS analysis of the evolved substance, 2-methylpyridine from the thermal decomposition of *fac*-Fe(dpa)Cl_3 at 230 °C.

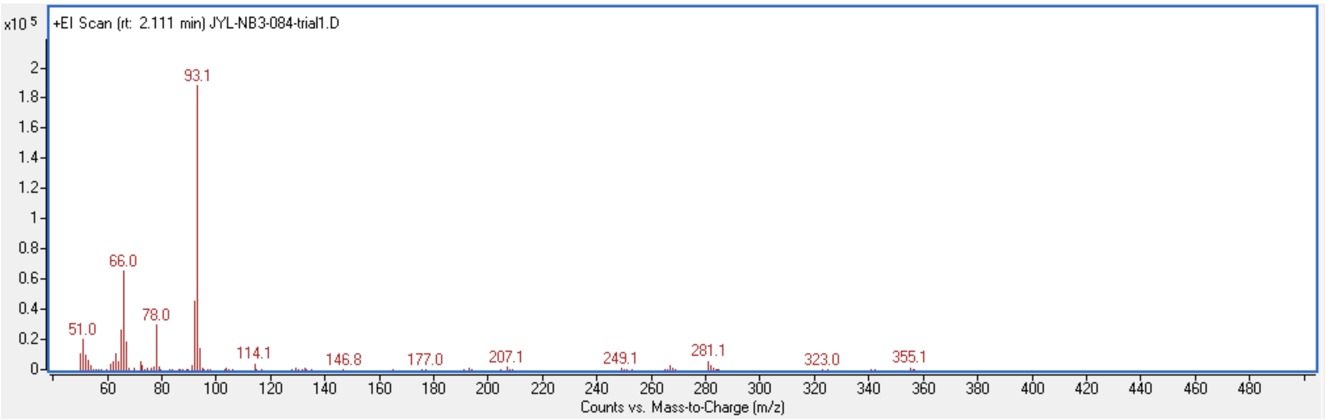

Figure S32. Mass fragmentation of the evolved substance, 2-methylpyridine at 2.111 minutes.

VI. IR Spectroscopy.

Table S2. Summary of IR characterization data for the solid produced from stirring $FeCl_3$ and dpa in different solvents and different temperatures. Match refers to those samples that are consistent with *fac*-Fe(dpa)Cl_3 spectra reported in the literature. Mismatch refers to those samples that form distinct Fe(dpa) complexes that were not further characterized.

Temp	MeOH	MeCN	THF	Toluene	DCE	EtOAc	DMC	DMF
rt	match	match	mismatch	match	mismatch	match	match	match
60 °C	match	match	mismatch	match	mismatch	match	match	match

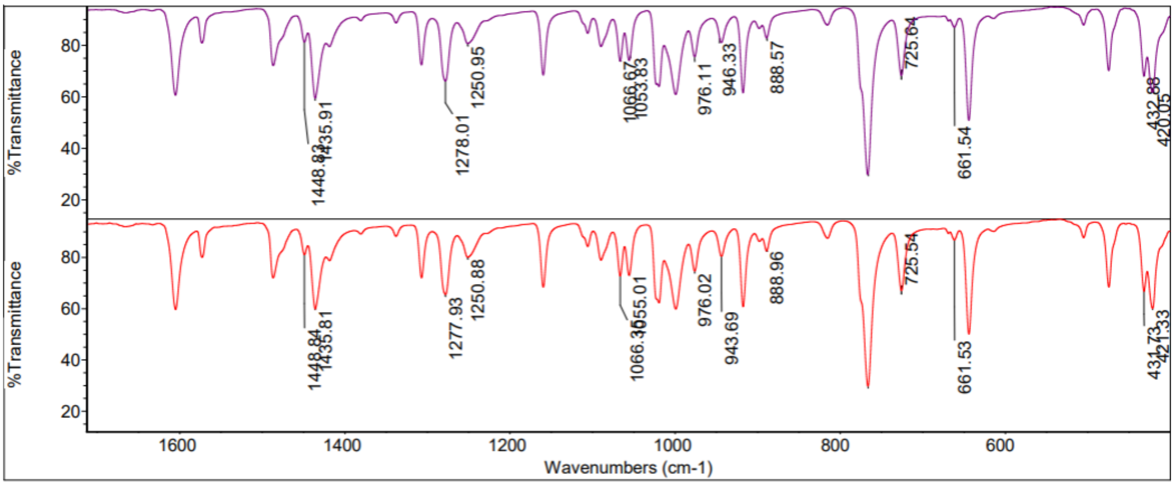

Figure S33. IR spectrum solid produced from stirring $FeCl_3$ and dpa in MeOH at rt (top) and IR spectrum of solid produced from stirring $FeCl_3$ and dpa in MeOH at 60 °C (bottom).

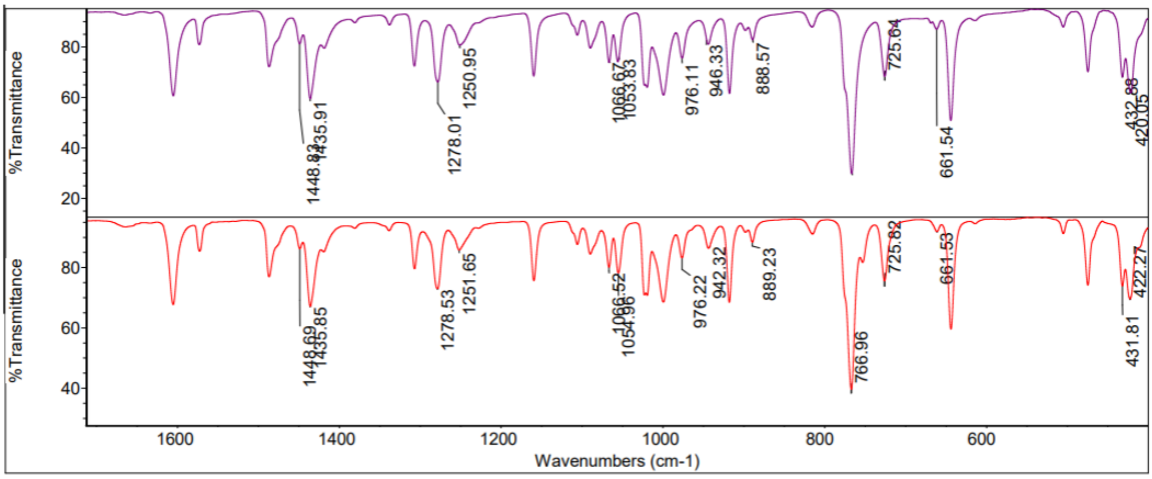

Figure S34. IR spectrum solid produced from stirring $FeCl_3$ and dpa in MeOH at 25 °C (top) and IR spectrum of solid produced from stirring $FeCl_3$ and dpa in MeCN at 25 °C.

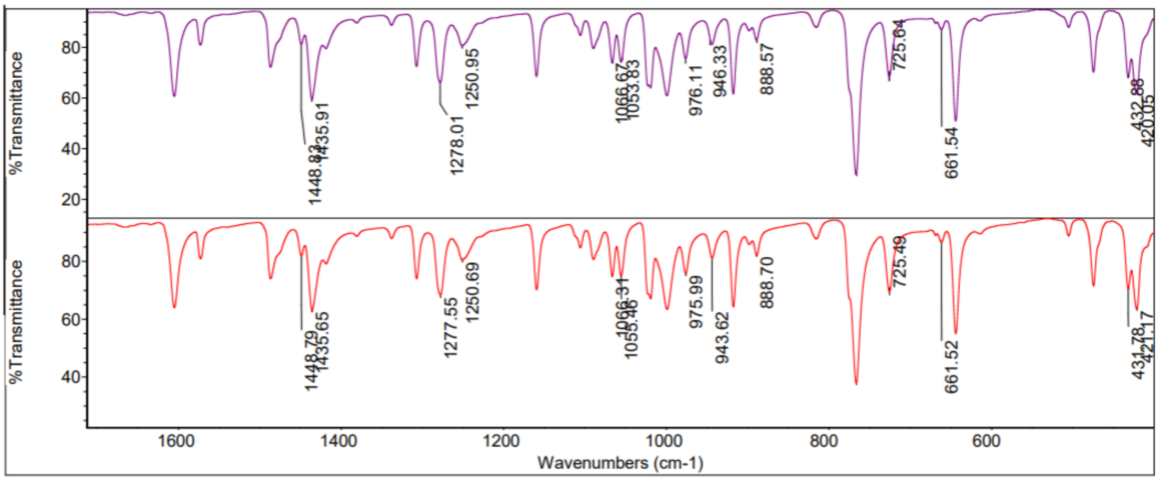

Figure S35. IR spectrum solid produced from stirring $FeCl_3$ and dpa in MeOH at 25 °C (top) and IR spectrum of solid produced from stirring $FeCl_3$ and dpa in MeCN at 60 °C.

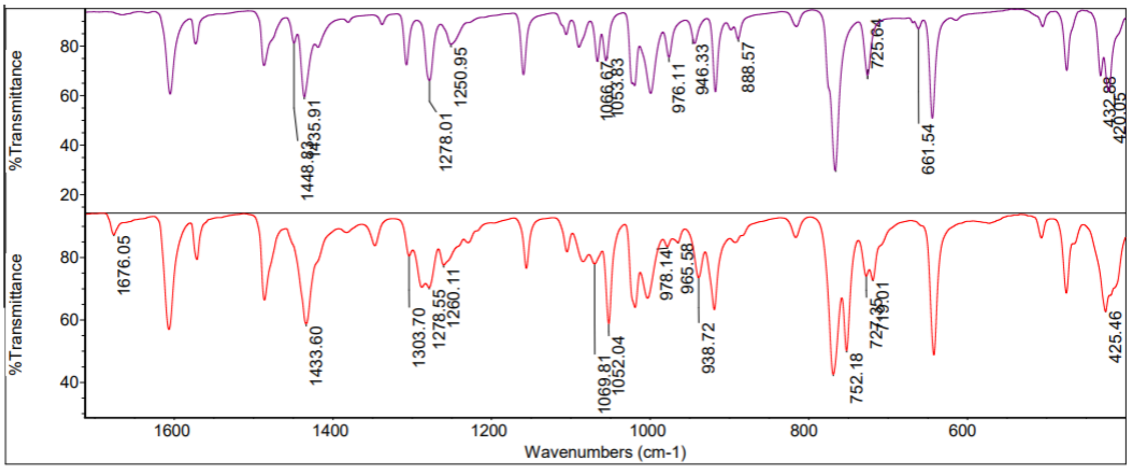

Figure S36. IR spectrum solid produced from stirring $FeCl_3$ and dpa in MeOH at 25 °C (top) and IR spectrum of solid produced from stirring $FeCl_3$ and dpa in THF at 25 °C.

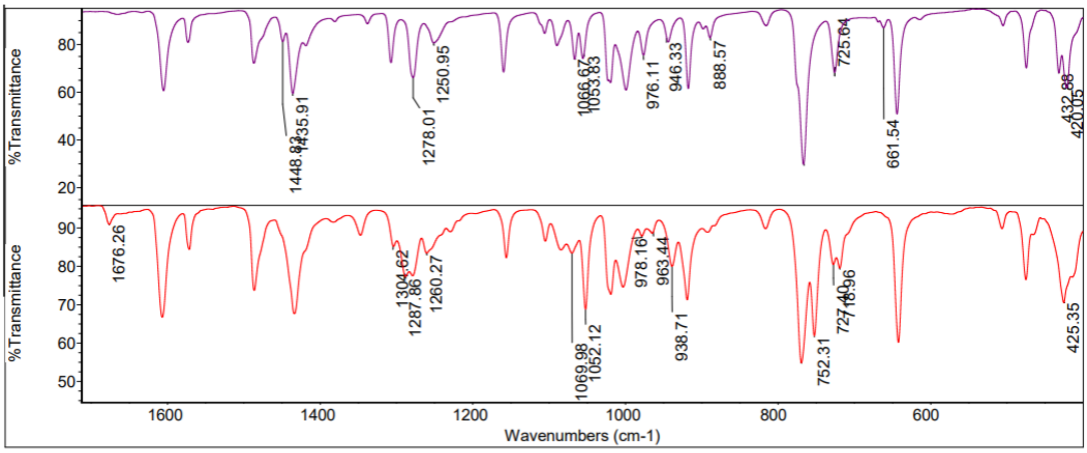

Figure S37. IR spectrum solid produced from stirring $FeCl_3$ and dpa in MeOH at 25 °C (top) and IR spectrum of solid produced from stirring $FeCl_3$ and dpa in THF at 60 °C.

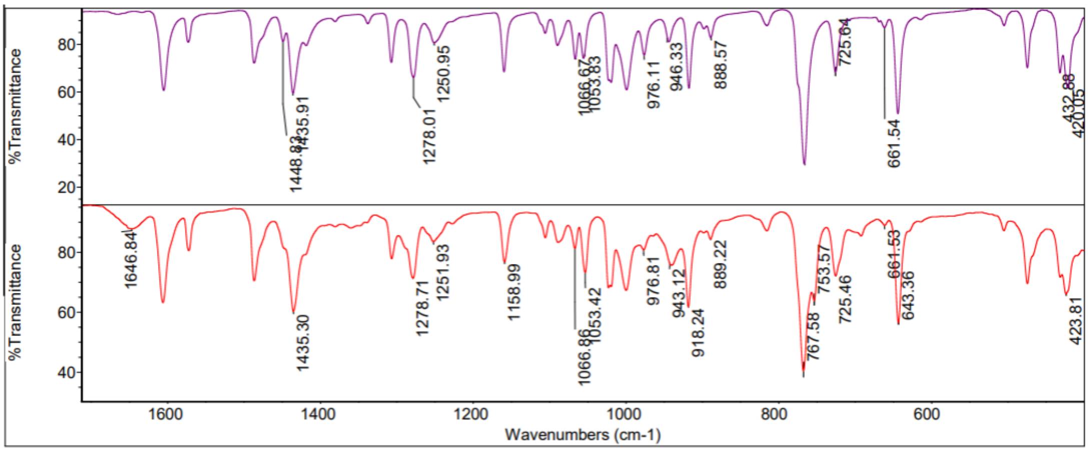

Figure S38. IR spectrum solid produced from stirring $FeCl_3$ and dpa in MeOH at 25 °C (top) and IR spectrum of solid produced from stirring $FeCl_3$ and dpa in toluene at 25 °C.

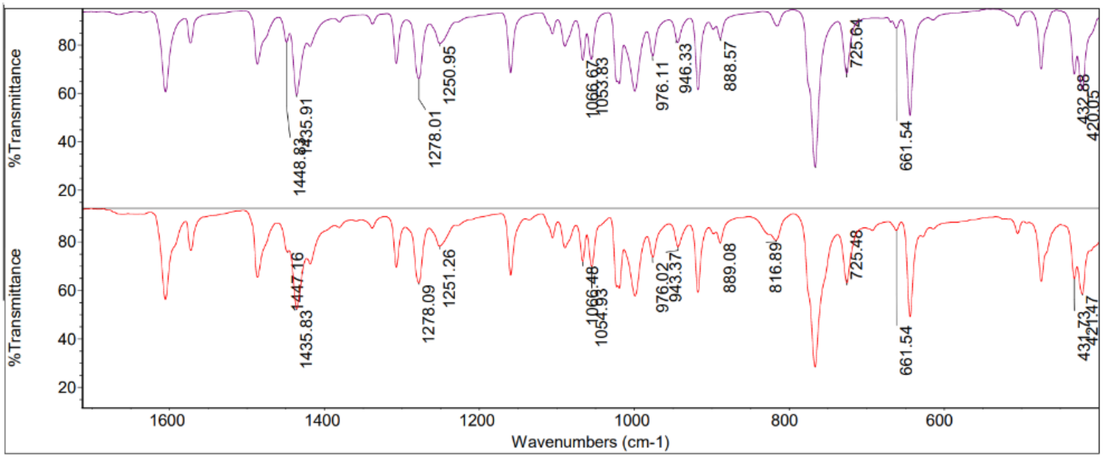

Figure S39. IR spectrum solid produced from stirring $FeCl_3$ and dpa in MeOH at 25 °C (top) and IR spectrum of solid produced from stirring $FeCl_3$ and dpa in toluene at 60 °C (bottom).

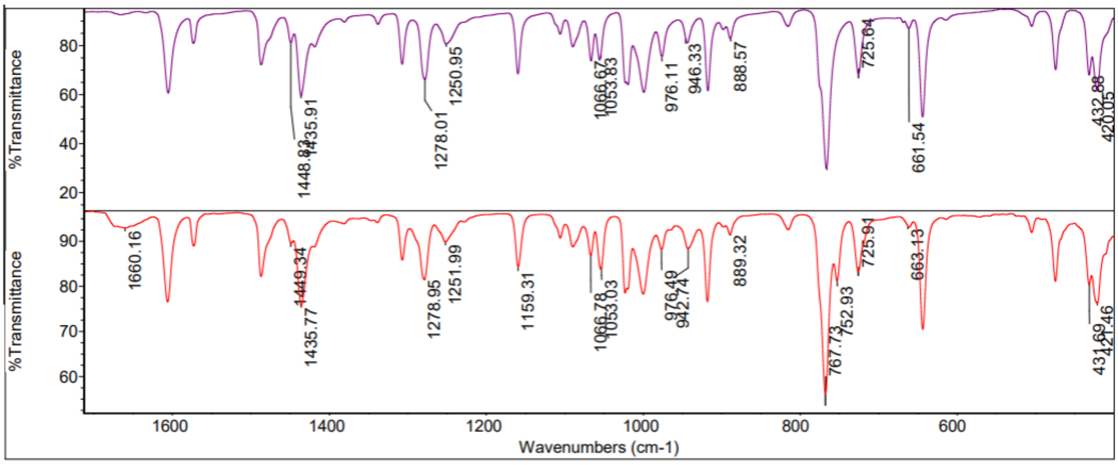

Figure S40. IR spectrum solid produced from stirring $FeCl_3$ and dpa in MeOH at 25 °C (top) and IR spectrum of solid produced from stirring $FeCl_3$ and dpa in DCE at 25 °C (bottom).

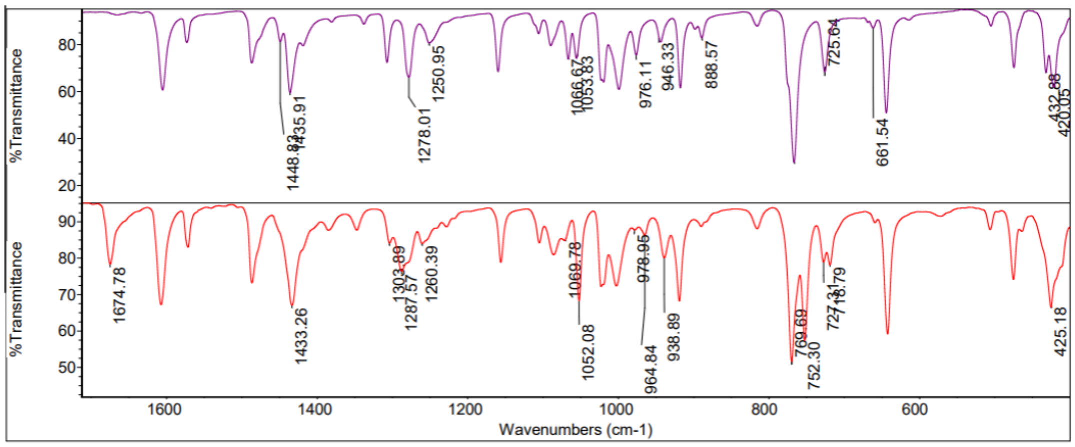

Figure S41. IR spectrum solid produced from stirring $FeCl_3$ and dpa in MeOH at 25 °C (top) and IR spectrum of solid produced from stirring $FeCl_3$ and dpa in DCE at 60 °C (bottom).

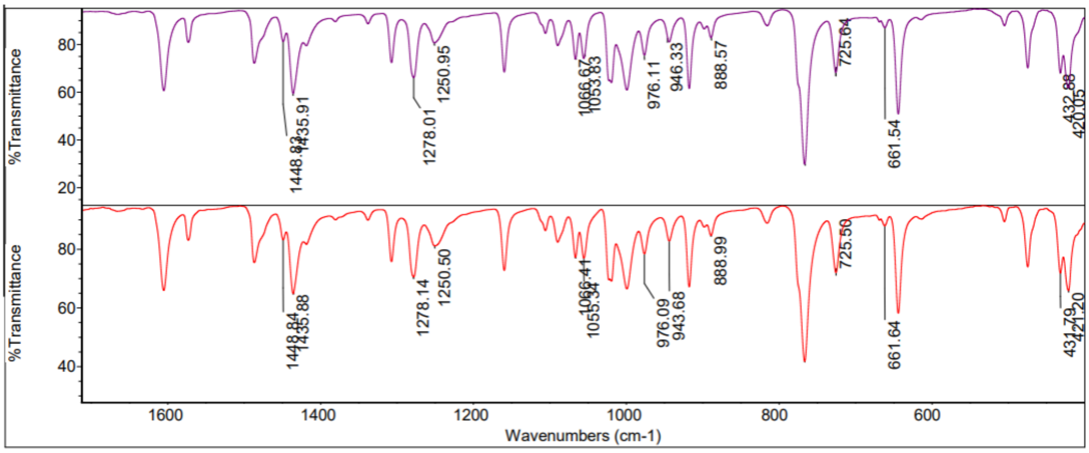

Figure S42. IR spectrum solid produced from stirring $FeCl_3$ and dpa in MeOH at 25 °C (top) and IR spectrum of solid produced from stirring $FeCl_3$ and dpa in EtOAc at 25 °C (bottom).

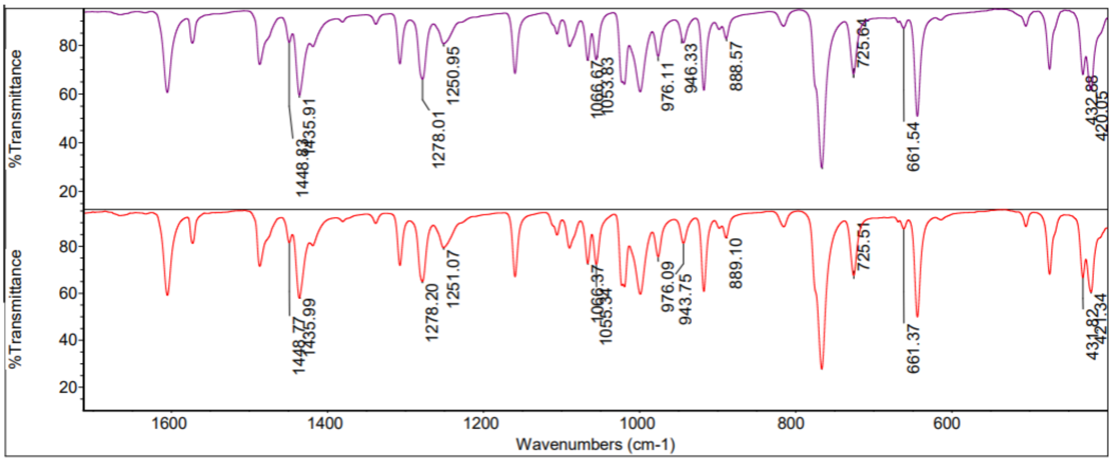

Figure S43. IR spectrum solid produced from stirring $FeCl_3$ and dpa in MeOH at 25 °C (top) and IR spectrum of solid produced from stirring $FeCl_3$ and dpa in EtOAc at 60 °C (bottom).

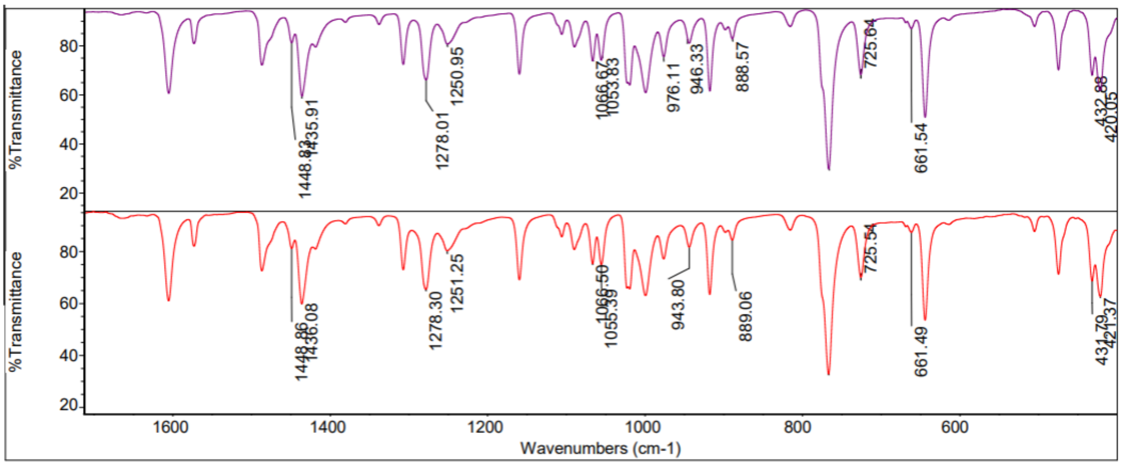

Figure S44. IR spectrum solid produced from stirring $FeCl_3$ and dpa in MeOH at 25 °C (top) and IR spectrum of solid produced from stirring $FeCl_3$ and dpa in DMC at 25 °C (bottom).

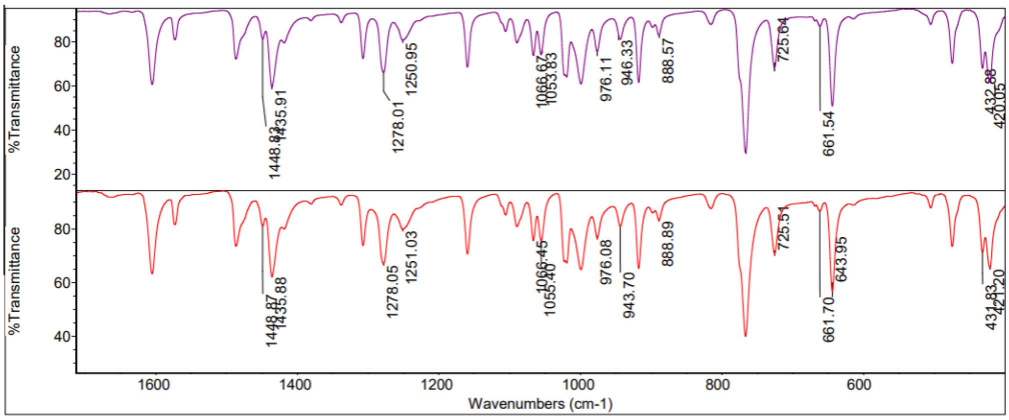

Figure S45. IR spectrum solid produced from stirring $FeCl_3$ and dpa in MeOH at 25 °C (top) and IR spectrum of solid produced from stirring $FeCl_3$ and dpa in DMC at 60 °C (bottom).

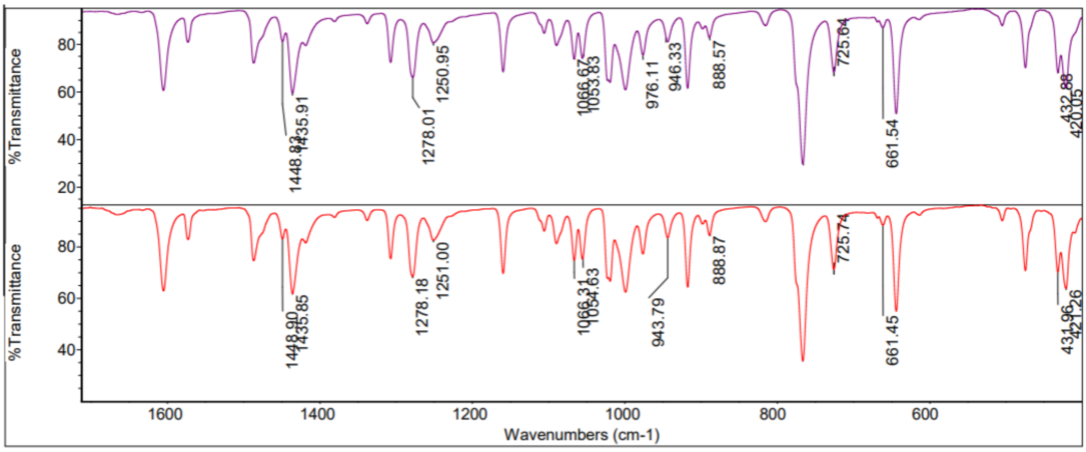

Figure S46. IR spectrum solid produced from stirring $FeCl_3$ and dpa in MeOH at 25 °C (top) and IR spectrum of solid produced from stirring $FeCl_3$ and dpa in DMF at 25 °C (bottom).

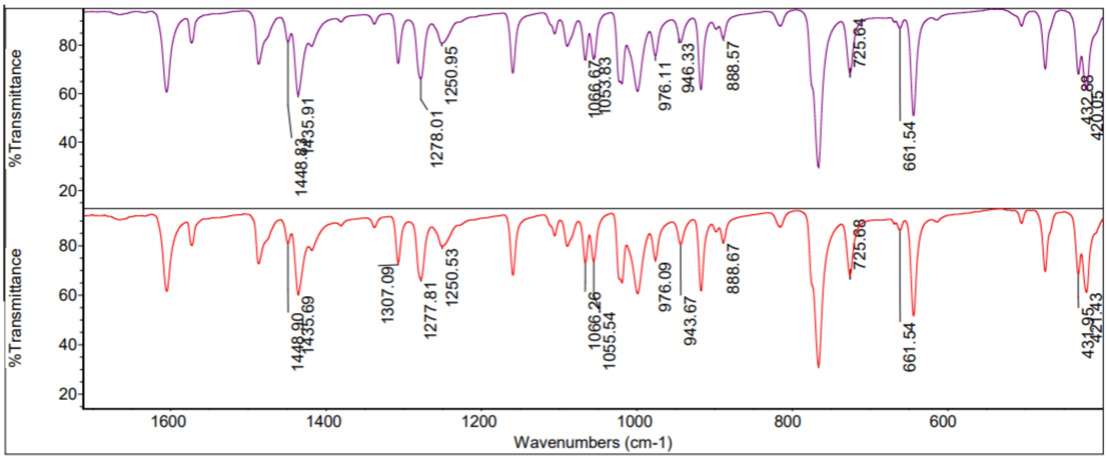

Figure S47. IR spectrum solid produced from stirring $FeCl_3$ and dpa in MeOH at 25 °C (top) and IR spectrum of solid produced from stirring $FeCl_3$ and dpa in DMF at 60 °C (bottom).

VII. Reaction Yields for Fe Mediated Decarboxylative Giese Reaction

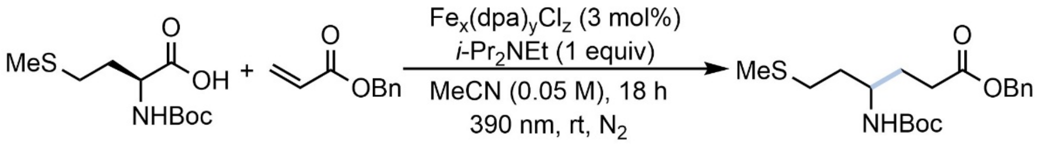

stirring $FeCl_3$ and dpa solvent	stirring at 25 °C yield	stirring at 60 °C yield	complex yield
toluene	0%	16%	27%
DMC	35%	40%	34%
EtOAc	48%	40%	34%
THF	38%	35%	32%
DCE	29%	24%	26%
MeOH	31%	29%	29%
DMF	35%	32%	34%
MeCN	23%	35%	32%
$[Fe(dpa)Cl_2][FeCl_4]$	-	-	41%
$[Fe(dpa)_2Cl_2][2CH_3OH]$	-	-	19%

Table S3. Reaction yields for Fe mediated decarboxylative Giese reaction using mixtures of $FeCl_3$ and dpa stirred in different solvents at 25 °C and 60 °C. $Fe(dpa)Cl_3$ complexes were also stirred in respective solvents at 25 °C and subjected to the Giese reaction.

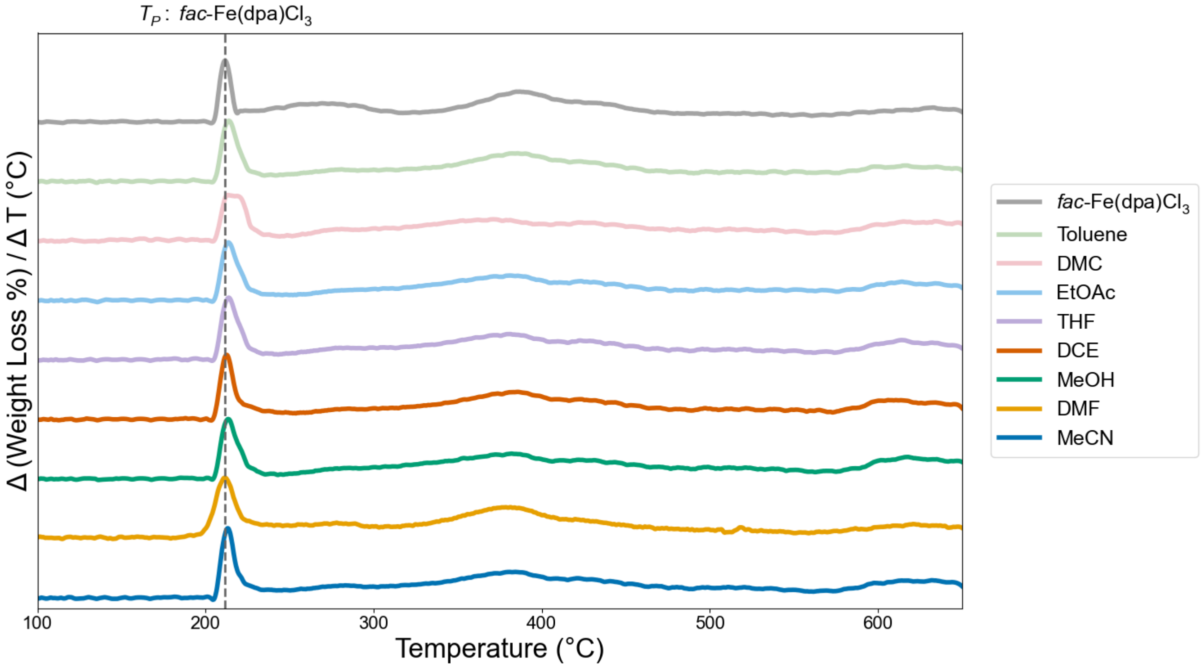

Figure S48. Comparison of TGA plots of *fac*-$Fe(dpa)Cl_3$ stirred in various solvents at 25 °C and submitted to TGA as aliquots.

VIII. Holding Temperature Analysis

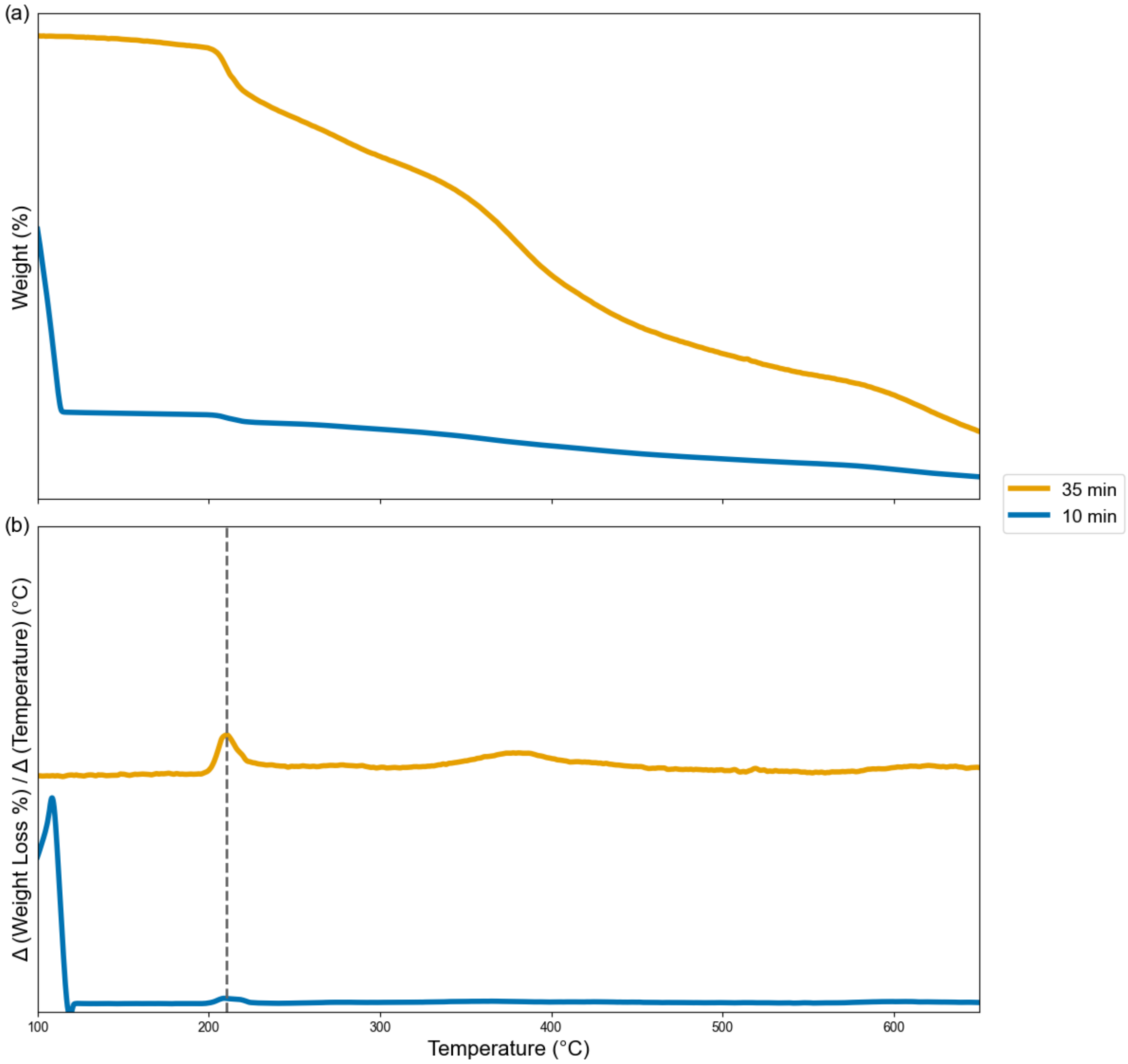

Figure S49. Analysis of two different holding temperatures at which the TGA was held at to account for DMF evolution were examined at 10 minutes and 35 minutes. Resolution of the TGA graph was seen to be higher when the hold temperature was held at 35 minutes to allow for full evolution of solvent.

IX. Percent Mass Loss for TGA Samples

Table S4. Percent mass loss as seen through TGA plots for respective samples.

Components	Solvent	Prestir Temperature (°C)	T_p (°C)	Weight loss % at T_p (%)
$Fe(dpa)Cl_3$	MeOH	25	213.48	7.67
$Fe(dpa)Cl_3$	MeCN	25	213.26	7.46
$Fe(dpa)Cl_3$	THF	25	213.85	8.14
$Fe(dpa)Cl_3$	Toluene	25	213.8	7.52
$Fe(dpa)Cl_3$	DCE	25	212.52	6.99
$Fe(dpa)Cl_3$	EtOAc	25	213.62	7.33
$Fe(dpa)Cl_3$	DMC	25	213.19	7.6
$Fe(dpa)Cl_3$	DMF	25	211.61	11.33
$Fe(dpa)Cl_3$	MeOH	60	214.74	7.8
$Fe(dpa)Cl_3$	MeCN	60	215.07	7.71
$Fe(dpa)Cl_3$	THF	60	214.05	6.66
$Fe(dpa)Cl_3$	Toluene	60	214.41	7.55
$Fe(dpa)Cl_3$	DCE	60	213.96	7.09
$Fe(dpa)Cl_3$	EtOAc	60	214.21	7.64
$Fe(dpa)Cl_3$	DMC	60	220.98	9.26
$Fe(dpa)Cl_3$	DMF	60	212.64	10.06
$FeCl_3$ + dpa	MeOH	25	210.58	10.06
$FeCl_3$ + dpa	MeCN	25	200.47	10.04
$FeCl_3$ + dpa	THF	25	223.76	13.77
$FeCl_3$ + dpa	Toluene	25	195.90	42.66*
$FeCl_3$ + dpa	DCE	25	204.78	16.81
$FeCl_3$ + dpa	EtOAc	25	188.90	10.56
$FeCl_3$ + dpa	DMC	25	222.22	13.04
$FeCl_3$ + dpa	DMF	25	210.57	10.5
$FeCl_3$ + dpa	MeOH	60	206.7	10.21
$FeCl_3$ + dpa	MeCN	60	202.05	16.39
$FeCl_3$ + dpa	THF	60	186.45	8.45*
$FeCl_3$ + dpa	Toluene	60	203.53	20.22
$FeCl_3$ + dpa	DCE	60	193.40	6.21*
$FeCl_3$ + dpa	EtOAc	60	188.88	14.90
$FeCl_3$ + dpa	DMC	60	217.25	13.23
$FeCl_3$ + dpa	DMF	60	218.14	10.74

X. X-ray data

Data were collected using a XtaLAB Synergy, Dualflex, and HyPix diffractometer equipped with an Oxford Cryosystems low-temperature device operating at T = 100.03(11) K. The structure was solved with the ShelXT 2018/2,[4] solution program using dual methods and by using Olex2 1.5-alpha as the graphical interface.[5] The model was refined with olex2. refine 1.5-alpha using full matrix least squares minimization on $\boldsymbol{F}^2$.[6]

1. X-ray crystal data of $[Fe(dpa)_2]Cl_2[2CH_3OH]$

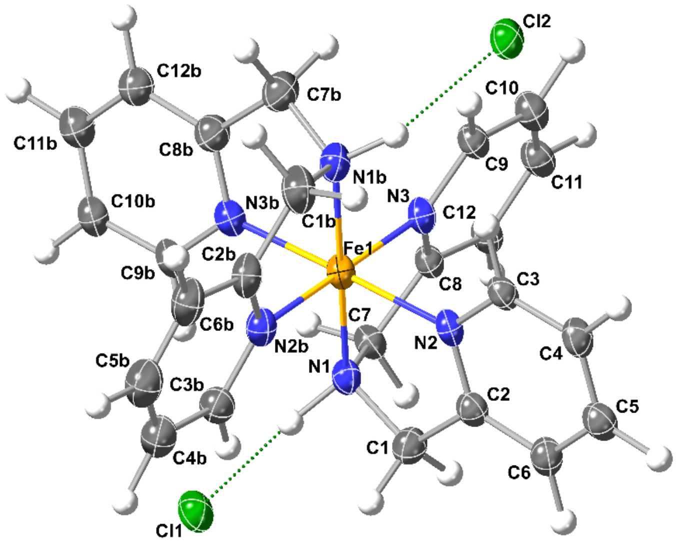

Figure S50. X-ray crystal structure for $[Fe(dpa)_2]Cl_2[2CH_3OH]$ with the atoms shown at thermal ellipsoids (50% probability level. There is one crystallographically independent molecule in the asymmetric unit, together with 2 methanol molecules (hidden for clarity). Single dark red block-shaped crystals of $[Fe(dpa)_2]Cl_2[2CH_3OH]$ were chosen from the sample as supplied. A suitable crystal with dimensions 0.30 × 0.24 × 0.13 mm was selected and mounted on a loop with paratone on a XtaLAB Synergy-S diffractometer. The crystal was kept at a steady T = 99.9(4) K during data collection.

**Table S5.** Crystal data and structure refinement for $[Fe(dpa)_2]Cl_2[2CH_3OH]$	
Compound	$[Fe(dpa)_2]Cl_2[2CH_3OH]$
Formula	$C_{26}H_{34}Cl_2FeN_6O_2$
$D_{calc.}$/ g cm^{-3}	1.441
m/mm^{-1}	6.553
Formula Weight	589.349
Colour	dark red
Shape	block-shaped
Size/mm	0.30×0.24×0.13
T/K	173.00(10)
Crystal System	triclinic
Space Group	*P*-1
a/Å	8.5845(3)
b/Å	9.0305(3)
c/Å	9.7308(3)
a/°	77.392(3)
b/°	83.794(3)
g/°	67.331(4)
V/Å^3	679.05(4)
Z	1
Z'	0.5
Wavelength/Å	1.54184
Radiation type	Cu K_a
Q_{min}/°	4.66
Q_{max}/°	77.47
Index range h	-10 ≥ h ≥ 10
Index range k	-11 ≥ k ≥ 10
Index range l	-11 ≥ l ≥ 12
Measured Refl's.	12311
Indep't Refl's	2774
Refl's I≥2*s*(I)	2762
R_{int}	0.0285
Parameters	315
Restraints	108
Largest Peak/eÅ^3	0.3488
Deepest Hole/eÅ^3	-0.3503
GooF	1.0918

R_1 (I≥2s(I) / all)	0.0211 / 0.0213
wR_2 (I≥2s(I) / all)	0.0532 / 0.0533

**Table S6.** Bond Lengths in Å for for $[Fe(dpa)_2]Cl_2[2CH_3OH]$		
**Atom**	**Atom**	**Length/Å**
C1	C2	1.4962(14)
C1	N1	1.4804(12)
C1	H1a	1.109(13)
C1	H1b	1.087(14)
C2	C6	1.3917(14)
C2	N2	1.3487(13)
C3	C4	1.3876(15)
C3	N2	1.3476(13)
C3	H3	1.085(13)
C4	C5	1.3894(17)
C4	H4	1.090(14)
C5	C6	1.3888(16)
C5	H5	1.087(14)
C6	H6	1.091(14)
C7	C8	1.4925(15)
C7	N1	1.4803(13)
C7	H7a	1.083(15)
C7	H7b	1.105(14)
C8	C12	1.3875(15)
C8	N3	1.3503(13)
C9	C10	1.3878(15)
C9	N3	1.3460(13)
C9	H9	1.107(13)
C10	C11	1.3880(17)
C10	H10	1.116(16)
C11	C12	1.3870(17)
C11	H11	1.050(15)
C12	H12	1.084(15)
Fe1	N1	2.0381(8)
Fe1	N1[1]	2.0381(8)
Fe1	N2	1.9925(8)
Fe1	N2[1]	1.9925(8)
Fe1	N3[1]	1.9978(8)

Fe1	N3	1.9978(8)
N1	H1	1.022(14)
C1s	O1s	1.4024(17)
C1s	H1sa	1.051(11)
C1s	H1sb	1.051(11)
C1s	H1sc	1.051(11)
O1s	H1s	0.96(2)

Table S7. Bond Angles in ° for $[Fe(dpa)_2]Cl_2[2CH_3OH]$

Atom	Atom	Atom	Angle/°
N1	C1	C2	110.97(8)
H1a	C1	C2	111.1(8)
H1a	C1	N1	110.8(8)
H1b	C1	C2	108.2(8)
H1b	C1	N1	108.4(8)
H1b	C1	H1a	107.3(12)
C6	C2	C1	121.96(9)
N2	C2	C1	115.33(8)
N2	C2	C6	122.68(9)
N2	C3	C4	122.55(10)
H3	C3	C4	120.9(7)
H3	C3	N2	116.5(7)
C5	C4	C3	118.99(11)
H4	C4	C3	119.8(8)
H4	C4	C5	121.2(8)
C6	C5	C4	118.99(10)
H5	C5	C4	121.2(9)
H5	C5	C6	119.8(9)
C5	C6	C2	118.66(10)
H6	C6	C2	118.5(8)
H6	C6	C5	122.8(8)
N1	C7	C8	110.53(8)
H7a	C7	C8	108.4(9)
H7a	C7	N1	108.5(9)
H7b	C7	C8	109.9(8)
H7b	C7	N1	112.5(8)
H7b	C7	H7a	106.9(12)
C12	C8	C7	121.89(10)
N3	C8	C7	115.55(9)

N3	C8	C12	122.56(10)
N3	C9	C10	122.62(10)
H9	C9	C10	120.0(7)
H9	C9	N3	117.4(7)
C11	C10	C9	119.36(11)
H10	C10	C9	118.7(8)
H10	C10	C11	121.9(8)
C12	C11	C10	118.24(11)
H11	C11	C10	122.2(9)
H11	C11	C12	119.4(9)
C11	C12	C8	119.36(11)
H12	C12	C8	119.3(8)
H12	C12	C11	121.3(8)
$N1^1$	Fe1	N1	180.0
$N2^1$	Fe1	N1	96.73(3)
N2	Fe1	N1	83.27(3)
N2	Fe1	$N1^1$	96.73(3)
$N2^1$	Fe1	$N1^1$	83.27(3)
$N2^1$	Fe1	N2	180.0
N3	Fe1	$N1^1$	97.17(3)
$N3^1$	Fe1	N1	97.17(3)
N3	Fe1	N1	82.83(3)
$N3^1$	Fe1	$N1^1$	82.83(3)
N3	Fe1	$N2^1$	95.00(3)
$N3^1$	Fe1	N2	95.00(3)
$N3^1$	Fe1	$N2^1$	85.00(3)
N3	Fe1	N2	85.00(3)
N3	Fe1	$N3^1$	180.0
C7	N1	C1	112.41(8)
Fe1	N1	C1	108.70(6)
Fe1	N1	C7	108.81(6)
H1	N1	C1	106.1(7)
H1	N1	C7	105.9(8)
H1	N1	Fe1	114.9(8)
C3	N2	C2	118.10(9)
Fe1	N2	C2	114.69(7)
Fe1	N2	C3	126.48(7)
C9	N3	C8	117.80(9)
$Fe1^1$	N3	C8	114.61(7)

Fe1[1]	N3	C9	126.93(7)
H1sa	C1s	O1s	109.5
H1sb	C1s	O1s	109.5
H1sb	C1s	H1sa	109.5
H1sc	C1s	O1s	109.5
H1sc	C1s	H1sa	109.5
H1sc	C1s	H1sb	109.5
H1s	O1s	C1s	108.4(11)

Table S8. Torsion Angles in ° for [Fe(dpa)$_2$]Cl$_2$[2CH$_3$OH]

**Atom**	**Atom**	**Atom**	**Atom**	**Angle/°**
C1	C2	C6	C5	-176.95(10)
C1	C2	N2	C3	176.95(9)
C1	C2	N2	Fe1	-12.22(9)
C1	N1	C7	C8	-90.97(9)
C2	C1	N1	C7	92.68(9)
C2	C1	N1	Fe1	-27.84(8)
C2	C6	C5	C4	0.06(12)
C2	N2	C3	C4	0.13(11)
C3	C4	C5	C6	-1.37(12)
C3	N2	C2	C6	-1.54(11)
C4	C3	N2	Fe1	-169.49(8)
C5	C4	C3	N2	1.31(13)
C5	C6	C2	N2	1.44(12)
C6	C2	C1	N1	-154.41(10)
C6	C2	N2	Fe1	169.29(8)
C7	C8	C12	C11	177.14(11)
C7	C8	N3	C9	-178.03(9)
C7	C8	N3	Fe1	10.64(10)
C8	C7	N1	Fe1	29.49(9)
C8	C12	C11	C10	0.61(13)
C8	N3	C9	C10	1.04(10)
C9	C10	C11	C12	1.48(13)
C9	N3	C8	C12	1.19(11)
C10	C9	N3	Fe1	171.17(8)
C11	C10	C9	N3	-2.39(13)
C11	C12	C8	N3	-2.03(14)
C12	C8	C7	N1	153.66(11)
C12	C8	N3	Fe1	-170.14(9)

N1	C1	C2	N2	27.09(9)
N1	C7	C8	N3	-27.11(10)

**Table S9.** Hydrogen Bond information for $[Fe(dpa)_2]Cl_2[2CH_3OH]$						
**D**	**H**	**A**	**d(D-H)/Å**	**d(H-A)/Å**	**d(D-A)/Å**	**D-H-A/deg**
C4	H4	O1s[1]	1.090(14)	2.378(14)	3.3679(16)	150.1(11)
N1	H1	Cl1	1.022(14)	2.194(14)	3.1856(9)	162.9(11)
O1s	H1s	Cl1	0.96(2)	2.18(2)	3.1247(11)	171(2)

[1]-x,1-y,1-z

2. X-ray crystal data of [Fe(dpa)Cl_2]$FeCl_4$

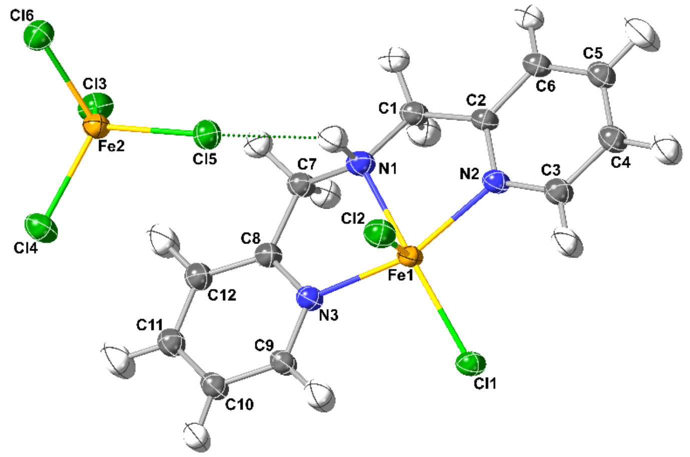

Figure S51. X-ray crystal structure for [Fe(dpa)Cl_2][$FeCl_4$] with the atoms shown at thermal ellipsoids (50% probability level. There is one crystallographically independent molecule in the asymmetric unit. Single light-yellow plate-shaped crystals of dpa$FeCl_2FeCl_4$ were chosen from the sample as supplied. A suitable crystal with dimensions 0.08 × 0.07 × 0.04 mm was selected and mounted on a loop with paratone on a XtaLAB Synergy, Dualflex, HyPix diffractometer. The crystal was kept at a steady T = 100.00(10) K during data collection.

**Table S10**. Crystal data and structure refinement for [Fe(dpa)Cl_2][$FeCl_4$].	
Compound	[Fe(dpa)Cl_2][$FeCl_4$]
Formula	$C_{12}H_{13}Cl_6Fe_2N_3$
$D_{calc.}$/ g cm^{-3}	1.831
m/mm^{-1}	20.014
Formula Weight	523.663
Colour	light yellow
Shape	plate-shaped
Size/mm	0.08×0.07×0.04
T/K	100.00(10)
Crystal System	monoclinic
Space Group	$P2_1/n$
a/Å	7.8734(2)
b/Å	10.4700(2)
c/Å	23.0500(4)
a/°	90
b/°	90.889(2)
g/°	90
V/Å^3	1899.89(7)

Z	4
Z'	1
Wavelength/Å	1.54184
Radiation type	Cu K_a
$Q_{min}/^{\circ}$	3.84
$Q_{max}/^{\circ}$	77.49
Measured Refl's.	17758
Indep't Refl's	3844
Refl's I≥2s(I)	3218
R_{int}	0.0573
Parameters	422
Restraints	228
Largest Peak/eÅ^3	0.7618
Deepest Hole/eÅ^3	-0.4322
GooF	1.0391
wR_2 (all data)	0.0670
wR_2	0.0632
R_1 (all data)	0.0424
R_1	0.0312

Table S11. Bond Lengths in Å for $[Fe(dpa)Cl_2][FeCl_4]$.

Atom	Atom	Length/Å
Fe1	Cl1[1]	2.6930(18)
Fe1	Cl1	2.2919(16)
Fe1	Cl2	2.2210(18)
Fe1	N1	2.138(2)
Fe1	N2	2.101(2)
Fe1	N3	2.094(2)
N1	H1	1.15(4)
N1	C1	1.467(3)
N1	C7	1.471(3)
N2	C2	1.347(3)
N2	C3	1.345(3)
N3	C8	1.337(3)
N3	C9	1.349(3)
C1	H1a	1.06(3)
C1	H1b	1.10(3)
C1	C2	1.504(4)

C2	C6	1.393(4)
C3	H3	1.11(3)
C3	C4	1.391(4)
C4	H4	1.15(3)
C4	C5	1.387(4)
C5	H5	1.06(3)
C5	C6	1.389(4)
C6	H6	1.07(3)
C7	H7a	1.09(3)
C7	H7b	1.10(3)
C7	C8	1.500(4)
C8	C12	1.398(4)
C9	H9	1.11(3)
C9	C10	1.383(4)
C10	H10	1.08(3)
C10	C11	1.387(4)
C11	H11	1.10(3)
C11	C12	1.384(4)
C12	H12	1.10(3)
Fe2	Cl3	2.2057(12)
Fe2	Cl4	2.1864(12)
Fe2	Cl5	2.1970(11)
Fe2	Cl6	2.1812(11)

**Table S12.** Bond Angles in ° for $[Fe(dpa)Cl_2][FeCl_4]$.			
**Atom**	**Atom**	**Atom**	**Angle/°**
Cl2	Fe1	Cl1	98.69(6)
Cl2	Fe1	Cl1[1]	178.15(7)
N1	Fe1	Cl1	162.59(9)
N1	Fe1	Cl1[1]	82.19(8)
N1	Fe1	Cl2	98.69(9)
N2	Fe1	Cl1[1]	82.84(8)
N2	Fe1	Cl1	101.33(8)
N2	Fe1	Cl2	95.74(8)
N2	Fe1	N1	77.84(9)
N3	Fe1	Cl1	98.89(8)
N3	Fe1	Cl1[1]	85.12(8)
N3	Fe1	Cl2	96.64(8)

N3	Fe1	N1	78.13(9)
N3	Fe1	N2	154.34(9)
H1	N1	Fe1	100.0(17)
C1	N1	Fe1	112.29(17)
C1	N1	H1	111.7(18)
C7	N1	Fe1	111.51(16)
C7	N1	H1	104.8(18)
C7	N1	C1	115.3(2)
C2	N2	Fe1	116.81(17)
C3	N2	Fe1	122.78(18)
C3	N2	C2	120.3(2)
C8	N3	Fe1	116.73(17)
C9	N3	Fe1	123.29(17)
C9	N3	C8	119.8(2)
H1a	C1	N1	112.1(17)
H1b	C1	N1	107.0(17)
H1b	C1	H1a	106(2)
C2	C1	N1	110.2(2)
C2	C1	H1a	113.8(17)
C2	C1	H1b	107.4(17)
C1	C2	N2	116.5(2)
C6	C2	N2	120.9(2)
C6	C2	C1	122.5(2)
H3	C3	N2	119.6(16)
C4	C3	N2	121.6(3)
C4	C3	H3	118.8(16)
H4	C4	C3	120.8(16)
C5	C4	C3	118.5(3)
C5	C4	H4	120.8(16)
H5	C5	C4	120.3(19)
C6	C5	C4	119.9(3)
C6	C5	H5	119.8(19)
C5	C6	C2	118.9(2)
H6	C6	C2	120.1(16)
H6	C6	C5	121.0(16)
H7a	C7	N1	107.2(16)
H7b	C7	N1	114.8(16)
H7b	C7	H7a	108(2)
C8	C7	N1	110.2(2)

C8	C7	H7a	108.5(16)
C8	C7	H7b	108.2(16)
C7	C8	N3	116.8(2)
C12	C8	N3	121.2(2)
C12	C8	C7	121.9(2)
H9	C9	N3	117.0(16)
C10	C9	N3	121.5(2)
C10	C9	H9	121.5(16)
H10	C10	C9	117.2(16)
C11	C10	C9	119.4(3)
C11	C10	H10	123.3(16)
H11	C11	C10	122.7(18)
C12	C11	C10	118.8(3)
C12	C11	H11	118.4(18)
C11	C12	C8	119.3(2)
H12	C12	C8	122.2(17)
H12	C12	C11	118.4(17)
Cl4	Fe2	Cl3	110.09(5)
Cl5	Fe2	Cl3	112.03(5)
Cl5	Fe2	Cl4	107.04(4)
Cl6	Fe2	Cl3	107.22(4)
Cl6	Fe2	Cl4	109.61(5)
Cl6	Fe2	Cl5	110.86(5)

Table S13. Torsion Angles in ° for dpa$FeCl_2FeCl_4$.

**Atom**	**Atom**	**Atom**	**Atom**	**Angle/°**
Fe1	N1	C1	C2	-27.65(18)
Fe1	N1	C7	C8	28.04(19)
Fe1	N2	C2	C1	-5.2(2)
Fe1	N2	C2	C6	176.17(19)
Fe1	N2	C3	C4	-175.6(2)
Fe1	N3	C8	C7	5.9(2)
Fe1	N3	C8	C12	-176.53(19)
Fe1	N3	C9	C10	175.2(2)
N1	C1	C2	N2	21.9(3)
N1	C1	C2	C6	-159.5(2)
N1	C7	C8	N3	-22.8(3)

N1	C7	C8	C12	159.7(2)
N2	C2	C6	C5	-0.8(3)
N2	C3	C4	C5	0.1(3)
N3	C8	C12	C11	1.6(3)
N3	C9	C10	C11	0.4(3)
C1	N1	C7	C8	157.6(2)
C1	C2	N2	C3	178.9(2)
C1	C2	C6	C5	-179.4(3)
C2	N2	C3	C4	0.1(3)
C2	C1	N1	C7	-156.8(2)
C2	C6	C5	C4	1.0(3)
C3	N2	C2	C6	0.3(3)
C3	C4	C5	C6	-0.7(3)
C7	C8	N3	C9	-179.3(2)
C7	C8	C12	C11	179.1(3)
C8	N3	C9	C10	0.7(3)
C8	C12	C11	C10	-0.4(3)
C9	N3	C8	C12	-1.7(3)
C9	C10	C11	C12	-0.5(3)

XI. References.

(1) Gottlieb, H. E.; Kotlyar, V.; Nudelman, A. NMR Chemical Shifts of Common Laboratory Solvents as Trace Impurities. *J. Org. Chem.* **1997**, *62* (21), 7512–7515. https://doi.org/10.1021/jo971176v.

(2) Fulmer, G. R.; Miller, A. J. M.; Sherden, N. H.; Gottlieb, H. E.; Nudelman, A.; Stoltz, B. M.; Bercaw, J. E.; Goldberg, K. I. NMR Chemical Shifts of Trace Impurities: Common Laboratory Solvents, Organics, and Gases in Deuterated Solvents Relevant to the Organometallic Chemist. *Organometallics* **2010**, *29* (9), 2176–2179. https://doi.org/10.1021/om100106e.

(3) Viswanathan, R.; Palaniandavar, M.; Balasubramanian, T.; Muthiah, P. T. Synthesis, Structure, Spectra and Redox Chemistry of Iron(III) Complexes of Tridentate Pyridyl and Benzimidazolyl Ligands. *J. Chem. Soc., Dalton Trans.* **1996**, No. 12, 2519–2525. https://doi.org/10.1039/DT9960002519.

(4) Sheldrick, G. M. Crystal Structure Refinement with SHELXL. *Acta Cryst C* **2015**, *71* (1), 3–8. https://doi.org/10.1107/S2053229614024218.

(5) Dolomanov, O. V.; Bourhis, L. J.; Gildea, R. J.; Howard, J. a. K.; Puschmann, H. OLEX2: A Complete Structure Solution, Refinement and Analysis Program. *J Appl Cryst* **2009**, *42* (2), 339–341. https://doi.org/10.1107/S0021889808042726.

(6) Bourhis, L. J.; Dolomanov, O. V.; Gildea, R. J.; Howard, J. A. K.; Puschmann, H. The Anatomy of a Comprehensive Constrained, Restrained Refinement Program for the Modern Computing Environment – Olex2 Dissected. *Acta Crystallogr A Found Adv* **2015**, *71* (Pt 1), 59–75. https://doi.org/10.1107/S2053273314022207.
